# The evolution of spectrum in antibiotics and bacteriocins

**DOI:** 10.1073/pnas.2205407119

**Published:** 2022-09-13

**Authors:** Jacob D. Palmer, Kevin R. Foster

**Affiliations:** ^a^Department of Biology, University of Oxford, Oxford, OX1 3RB, United Kingdom;; ^b^Department of Biochemistry, University of Oxford, Oxford, OX1 3QU, United Kingdom

**Keywords:** antibiotics, microbiology, evolution, spectrum, bacteriocin

## Abstract

A key property of many antibiotics is that they will kill or inhibit a diverse range of microbial species. This broad-spectrum of activity has its evolutionary roots in ecological competition, whereby bacteria and other microbes use antibiotics to suppress other strains and species. However, many bacteria also use narrow-spectrum toxins, such as bacteriocins, that principally target conspecifics. Why has such a diversity in spectrum evolved? Here, we develop an evolutionary model to understand antimicrobial spectrum. Our first model recapitulates the intuition that broad-spectrum is best, because it enables a microbe to kill a wider diversity of competitors. However, this model neglects an important property of antimicrobials: They are commonly bound, sequestered, or degraded by the cells they target. Incorporating this toxin loss reveals a major advantage to narrow-spectrum toxins: They target the strongest ecological competitor and avoid being used up on less important species. Why then would broad-spectrum toxins ever evolve? Our model predicts that broad-spectrum toxins will be favored by natural selection if a strain is highly abundant and can overpower both its key competitor and other species. We test this prediction by compiling and analyzing a database of the regulation and spectrum of toxins used in inter-bacterial competition. This analysis reveals a strong association between broad-spectrum toxins and density-dependent regulation, indicating that they are indeed used when strains are abundant. Our work provides a rationale for why bacteria commonly evolve narrow-spectrum toxins such as bacteriocins and suggests that the evolution of antibiotics proper is a signature of ecological dominance.

## Significance

Antibiotics, like penicillin, treat a wide range of infections. This broad-spectrum of activity has its evolutionary roots in microbial warfare, where antibiotics can provide a competitive edge. But bacteria also make narrow-spectrum toxins, which presents a puzzle: Why not use broad-spectrum toxins to target more competitors? Using evolutionary modelling, we show that narrow-spectrum toxins help focus an attack on a key competitor, minimizing toxin loss to other targets. Broad-spectrum attacks only make sense when a microbe is abundant and can make a lot of toxin. We survey available data and find, as predicted, that broad-spectrum toxins are typically made by bacteria at high abundance. This suggests that antibiotics evolved in dominant microbes that could afford to take on diverse competitors.

It is nearly a century since penicillin was accidentally discovered on a bacterial culture plate contaminated with mold (1). What made penicillin such a revolutionary drug was its ability to kill a diverse range of bacterial species (1). This broad-spectrum of activity meant that it could be used to treat a wide range of infections, including ones where the causal agent was unknown (2). In the decades since, countless microbial toxins have been identified, purified, and characterized (3) based on their spectrum of activity, and yet we understand almost nothing about the evolution of this defining trait.

Many antibiotics were originally isolated from microbes or are modifications of microbial products (4). By analogy with their use in medicine, it might be assumed that antibiotics will benefit producers by allowing them to eliminate a wide range of species. *Streptomyces* bacteria, for example, make many antibiotics, including several structurally similar to penicillin, which eliminate competing bacteria and other organisms (5). However, bacteria also make a wide range of antimicrobial compounds that have a much narrower spectrum of activity, including many bacteriocins (6–8). For example, the best studied bacteriocins are the colicins—protein toxins made by *Escherichia coli* and related species—and these appear to principally target members of the same species or genus (6). Such examples raise the question, why evolve such narrow-spectrum toxins, given the potential benefits of broad-spectrum drugs that can eliminate a much wider range of competitors?

Several evolutionary studies have asked why bacteria make antibiotics and bacteriocins (9–16), but these have not tackled the question of why there is such striking diversity in spectrum. We decided, therefore, to develop an evolutionary model to study this problem. We focus on a strain of bacteria living in a diverse community and ask, does natural selection favor the use of a toxin that only targets closely related strains (narrow-spectrum) or one that also targets members of the community more widely (broad-spectrum)? Our first model follows the basic intuition that broad-spectrum toxins give the most benefit to producers because they kill the most competitors. However, we then show that this result rests upon an unrealistic assumption present in most previous models of toxin competition, namely, that toxins are not bound, degraded, or otherwise inactivated by target cells. Accounting for these processes reveals a clear advantage to narrow-spectrum toxins: They ensure toxins are used on the most important competitors and not wasted on less important strains. Why then do broad-spectrum toxins evolve at all? We find that highly abundant bacteria can benefit from broad-spectrum toxins, as these strains have the numbers to successfully overcome a wide range of competitors. We test our model with a meta-analysis of published studies on toxin regulation and spectrum, which suggests—as we predict—that broad-spectrum toxins are typically used when bacteria are at high abundance.

## Results and Discussion

### Model Overview.

**Fig. 1.**
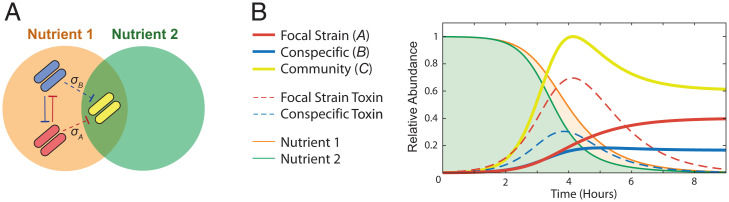
(*A*) Modeling scenario. The focal strain (red) produces a toxin that targets a conspecific strain (blue), with an evolvable spectrum (*σ_A_*) that means it may also target members of the wider community (yellow). The conspecific strain (blue) can also produce toxins that target the focal strain and the community. Members of the focal species (red and blue) compete over a limited nutrient (orange). The community has its own limiting nutrient (green), but there can be niche overlap with the focal species, defined by parameter (Ω_1_), leading to resource competition between the community and the focal species. (*B*) Sample dynamics from a single local competition between the focal strain (solid red line), a conspecific (solid blue line), and a community species (solid yellow line). The focal strain and the conspecific both produce toxins (dashed red and blue lines, respectively). The focal strain and conspecific can only consume nutrient 1 (orange), while the community consumes both nutrient 2 (green) and nutrient 1 (orange). For these illustrative plots, single-species abundances are normalized to a maximum abundance of 1.0, and combined toxin abundances are normalized to a maximum of 1.0, which allows the different dynamics to be easily seen. See *Methods* for details of model and parameters. In the plot, *σ_A_* = ,1.0; *σ_B_* = 1.0; *γ_A_* = 0.08; *γ_B_* = 0.03; *E* = 1, and other parameters are set to default values.

We are interested in the evolutionary impacts of a bacterium using broad- versus narrow-spectrum toxins to compete with other strains and species. We consider a focal species that can evolve to use toxins that 1) solely target members of the same species that share its ecological niche (narrow-spectrum) or 2) target these conspecifics and, additionally, members of the wider bacterial community (broad-spectrum) (Fig. 1). Specifically, we capture toxin spectrum with a variable *σ*, which lies on a continuum between a purely narrow-spectrum toxin (*σ* = 0) and an indiscriminate broad-spectrum toxin (*σ* = 1). We do not change the energetic cost of toxin production across spectrum values; that is, there is no spectrum trade-off built into the model.

The heart of the model is a system of differential equations that follow the focal strain, a conspecific strain, and the wider community as three parties that compete for resources in the environment. These ecological equations and their underlying assumptions closely follow established models of microbial competition (10–12, 14, 17, 18), and we use them to study the dynamics of the three parties, their toxins, and the two nutrients as they compete in a given patch. Because nutrients are limited, it follows that there can be an evolutionary incentive for the focal species to invest in toxins that suppress some, or all, of the other bacterial strains in the environment.

Following refs. 11 and 18, we predict evolutionary outcomes using evolutionary game theory in the tradition of Maynard Smith and Price and adaptive dynamics (19, 20), which identifies evolutionarily stable strategies (ESSs). With this information, we can then ask whether an established genotype of the focal species with toxin spectrum *σ* and production rate *γ* can be outcompeted by a new, rare genotype with a different strategy. More specifically, we assume that the bacteria live in a patchy landscape, where each patch could be within an individual host. Each competition is then modeled by the differential equations at the scale of one patch, but is assumed to be one of a large number that can occur in parallel. We then calculate fitness of a given strategy using an invasion analysis (20). This approach is detailed in *Methods* but is based on calculating the number of cells produced by the focal strain in its patch when using a rare, mutant strategy against a conspecific that uses the resident strategy and is currently established in the population. If the mutant strategy (e.g., using a broader-spectrum toxin than the resident) is predicted to invade, this strategy is then taken as the new resident strategy, and the process is iterated until an ESS is reached that cannot be invaded by other strategies. We do not model the evolution of toxin diversity here, which is an interesting topic in its own right, but it is commonly high in natural populations (9, 21, 22). Therefore, again following previous work (11, 23), we assume that toxin diversity is generally high, such that any two strains that meet will have toxins that they are able to use against each other.

The wider community in which a focal species lives will typically contain multiple species. To limit the model’s complexity, we capture this wider community with one differential equation, but the framework is extendable to include more species. The form of our models, which explicitly capture ecological dynamics, is not amenable to analytical treatment. Simpler models can always be considered if the priority is to achieve analytical tractability. However, such simplification is very limiting for models of toxin-mediated competition (see ref. 11 for a formal treatment of the limitations of analytical assessment in capturing toxin competition). Accordingly, here we follow previous work and use a numerical approach (10–12, 17). This methodology ensures our model is realistic enough to test its predictions using microbiological data, which is our priority (see *A Meta-analysis Suggests That Broad-Spectrum Toxins Are Deployed at High Cell Abundances*). We give more details of our approaches in *Methods*, and further background can be found in refs. 11 and 18.

### A Standard Model Predicts that Broad-Spectrum Toxins Will Always Evolve.

Our goal is to model how toxin spectrum and toxin production rate (i.e., level of investment) evolve for the focal species. In each competition, the focal strain must contend with another member of its species (conspecific)—which is competing for its specific metabolic niche—as well as the wider bacterial community (Fig. 1). As just discussed, our modeling assumptions and methods are based closely on previous theoretical work on the ecology and evolution of bacterial competition (*Methods*). However, our question needs us to also capture toxin spectrum, which lacks a precedent in the evolutionary literature. Here, based on the biochemistry of many toxins (24–27), we model the spectrum of activity as a toxin-receptor affinity problem using the Hill equation (15, 28). With a Hill coefficient of one, a single parameter, *K_ij_*, then determines toxin-receptor affinity for the toxin of species *i* for the receptor of target bacteria *j*. Toxin spectrum (*σ*) can then be defined as

$$\sigma_{i}=1-\left(\frac{K_{iC}-K_{\text{min}}}{K_{\text{max}}-K_{\text{min}}}\right),$$

where $K_{iC}$ defines the receptor affinity for the toxin of the focal species (*i*) toward members of the community (*C*) (note that a low *K* value in the Hill equation implies high affinity, and vice versa). The toxins always bind with the same affinity to the conspecific strain (*K_AB_* = *K_BA_* = *K*_min_ = 0.05). The equation then defines spectrum by comparing binding to the community (*K_iC_*) to the minimum (*K*_min_) and maximum (*K*_max)_ of the toxin across all targets. This puts *σ_i_* on a scale of zero to one. When *σ_i_* = 1, the toxin targets community members with equal affinity to the conspecific strain and is an indiscriminate broad-spectrum toxin (*K_AB_* = *K_BA_* = *K_iC_* = 0.05). When *σ_i_* = 0, the toxin is narrow-spectrum, targeting the conspecific strain with maximum affinity, and targeting the community with minimum affinity (*K_iC_* = *K*_max_ = 3.0).

A second novelty of our analysis is that we study the simultaneous evolution of two properties of toxins, both spectrum and production rate. To solve this two-parameter optimization problem, we first use game theory to find the production rate that is an evolutionary stable strategy (*γ_ESS_*) for all values of spectrum, when the two competing strains use toxins of equal spectrum (*σ_i_* = *σ_j_*). We then find the ESS for the spectrum (*σ_ESS_*), assuming that bacteria use the production rate ESS previously calculated for each spectrum value (*Methods* and *SI Appendix*, Fig. S1). In practice, this approach implies that bacteria can rapidly evolve to alter their production rate as the spectrum evolves. We believe this is a reasonable assumption, given the great flexibility in toxin regulation seen in bacteria (9, 11, 29, 30), and the marked changes in production rate that can occur from relatively minor mutational changes in regulatory regions(29). In summary, with this approach, the focal strain and the conspecific strain are coevolving both spectrum and production rate, while toxin diversity is assumed to be maintained at a high level in the population such that each strain will utilize a different toxin.

**Fig. 2.**
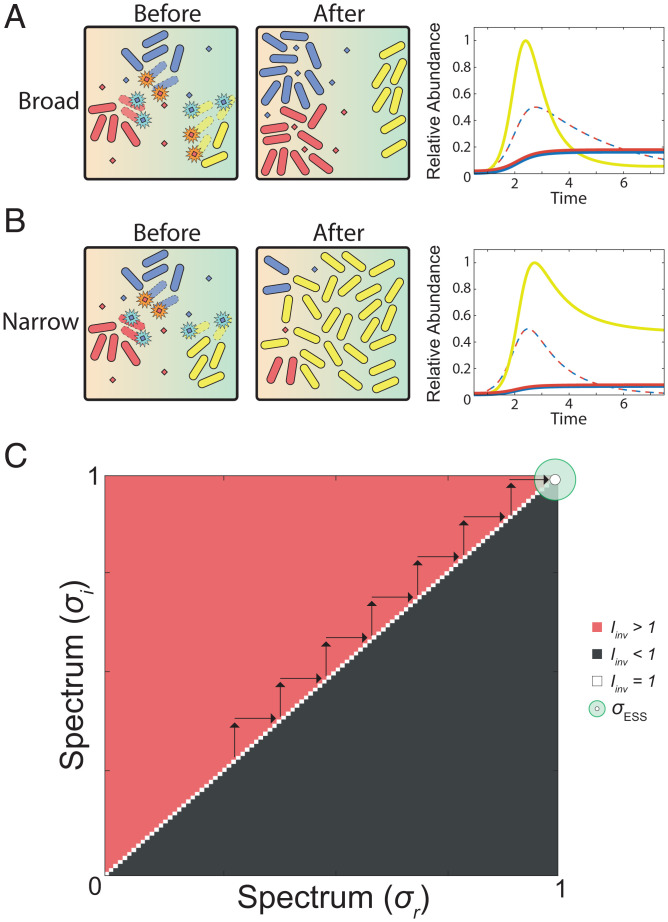
Broad-spectrum toxins evolve in a standard model of bacterial competition. (*A*) Broad-spectrum toxins allow a focal strain (red) to inhibit both its conspecific competitor (blue) and the community (yellow). Representative temporal dynamics are shown where the focal strain (red) and conspecific (blue) both produce broad-spectrum toxins (red and blue dashed lines, respectively) to inhibit each other, and the community (yellow). *σ_A_* = *σ_B_* = 1.0; γ*_A_* = γ*_B_* = 0.14027 (*γ_ESS_* for *σ* = 1.0); *E* = 5. (*B*) Narrow-spectrum toxins mean that the focal strain now fails to inhibit community growth as much. Even though the blue strain still makes a broad-spectrum toxin in this example, the result of narrow-spectrum toxin use by the red strain is that the community blooms and the abundance of both the red and blue strain falls. This outcome translates to lower fitness for the red strain from the use of narrow-spectrum toxins, as opposed to a broad-spectrum toxin. *σ_A_* = 0; *σ_B_* = 1.0; *γ_A_* = *γ_B_* = 0.14027 (*γ_ESS_* for *σ* = 1.0); *E* = 5. For these illustrative plots, single-species abundances are normalized to a maximum abundance of 1.0, and combined toxin abundances are normalized to a maximum of 1.0, which allows the different dynamics to be easily seen. (*C*) Pairwise invasibility plot for spectrum of activity (*σ*) using models of constant toxin degradation. The evolutionary stable strategy is indiscriminately broad (*σ_ESS_* = 1); *E* = 5. All parameters are default unless otherwise indicated.

This initial model predicts that the evolution of toxin spectrum is always a race for the broadest (Fig. 2*C*). That is, we find that a genotype carrying a narrower-spectrum toxin can always be outcompeted by one carrying a broader-spectrum toxin. The reason this occurs is that the broad-spectrum toxin is, in essence, better value. A broad-spectrum toxin allows a focal strain to inhibit both conspecifics and members of the wider community. When both conspecifics and the wider community compete for the focal strain’s limiting nutrient, this strategy carries evolutionary benefits over a narrow-spectrum toxin that only inhibits conspecifics. The additional benefit of broad-spectrum toxins that comes from suppressing the community is conferred on both the strain that makes the toxin and the conspecific strain (Fig. 2*A*). That is, it does not provide a specific advantage to the more broad-spectrum toxin producers within a given competition. However, as a result, a patch where the focal strain makes a broad-spectrum toxin (Fig. 2*A*) produces more cells of the focal strain than a patch where it makes a narrow-spectrum toxin (Fig. 2*B*). This patch-level effect gives an evolutionary benefit to broad-spectrum genotypes over narrow-spectrum ones.

### An Important Advantage to Narrow-Spectrum Toxins.

Our first model predicts that broad-spectrum toxins will always evolve. Not only does this prediction fly in the face of the biology of bacteriocins, which are often narrow-spectrum (6, 31, 32), but it also rests upon a potentially problematic assumption commonly used in previous studies of bacterial toxin competition (10–12, 14, 17). There, it has been typical to assume that toxins are lost at a constant rate, simply proportional to their concentration, and we followed this assumption with our first model. However, an alternative assumption is that toxins will be lost from the system in a cell density–dependent fashion, where loss rate is linked to the number of target cells in the system (13, 16, 33). While this results in a more complex model, there is a good reason to believe that this is the more realistic assumption. Many toxins are bound or otherwise trapped by their targets cells as a product of their inhibitory action, and toxins are also degraded (both inadvertently and via dedicated enzymes) (27, 34–36). In the terminology of game theory, these processes mean that toxins are diminishable as opposed to nondiminishable in our first model (37). In many models, the difference between these two assumptions may be a detail that has little impact on predictions. However, as we show below, for our question, this seemingly subtle distinction proves to be critical for predictions.

**Fig. 3.**
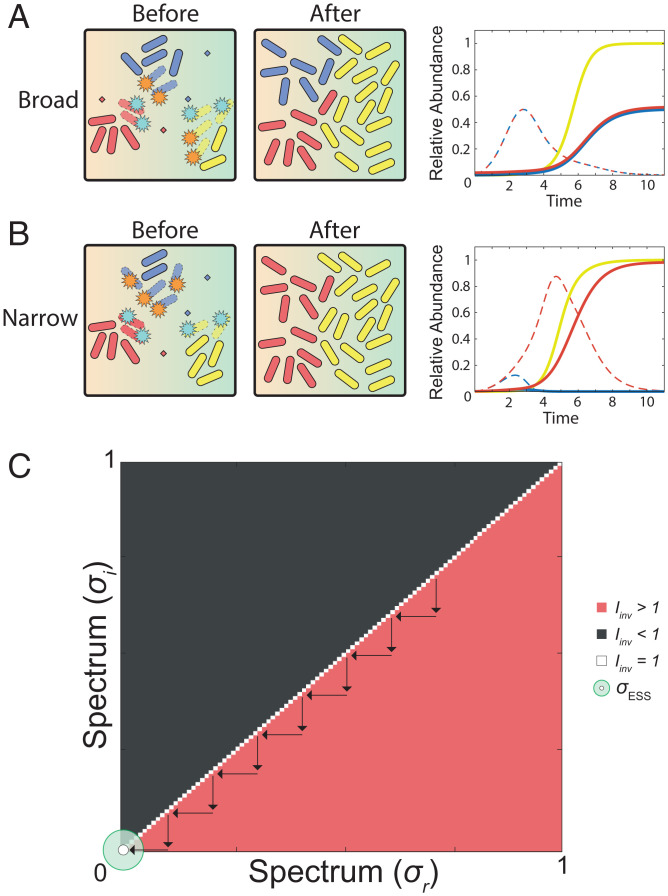
The advantage of narrow-spectrum toxins. In this model, we assume that toxin degradation or loss is caused by target cells (as opposed to simply assuming a constant loss rate; Fig. 2). (*A*) Broad-spectrum toxins are used by both the focal strain and the conspecific competitor. Large amounts of toxin are lost due to targeting the community (yellow), and toxin levels remain relatively low (dashed lines in dynamics plot) rendering them ineffective. The community has its own unique nutrient and grows fast enough to outpace toxin-mediated killing. *σ_A_* = *σ_B_* = 0.99; *γ_A_* = *γ_B_* = 0.16577 (*γ_ESS_* for *σ* = 0.99). (*B*) The focal strain produces a narrow-spectrum toxin, which allows it to outcompete the conspecific strain (blue), which is using a broad-spectrum toxin. The narrow-spectrum toxin experiences a much lower loss rate, allowing it to build up (red dashed line in dynamics plot) and give the focal strain its advantage. *σ_A_* = 0; *σ_B_* = 0.99; *γ_A_* = *γ_B_* = 0.22315 (*γ_ESS_* for *σ* = 0). For these illustrative plots, community abundance is normalized to a maximum abundance of 1.0 and combined focal species abundances and combined toxin abundances are normalized to a maximum of 1.0, which allows the different dynamics to be easily seen. (*C*) PIP for spectrum of activity (*σ*) using models of target density-dependent toxin degradation. The evolutionary stable spectrum strategy is fully narrow (*σ_ESS_* = 0). All parameters are default unless otherwise indicated.

We incorporated a model for toxin loss into our equations based upon the same Hill dynamics that we use to model spectrum of activity. With this change, the action of a toxin interacting with target cells is now linked to its removal from the system. Put another way, this captures the fact that toxins will be consumed or lost as a result of binding their targets and/or entering cells. This one change completely reverses the modeling predictions. Rather than always seeing natural selection for broad-spectrum toxins, we see the evolution of the narrowest possible toxin (Fig. 3*C*). Why does this occur? By incorporating toxin degradation processes, a major advantage of narrow-spectrum toxins becomes clear. Targeting other community members with a broad-spectrum toxin can be wasteful because it consumes toxin, but these species do not compete that strongly with the focal strain. By contrast, a narrow-spectrum toxin targets the most important competitors in the community, which compete directly for the same nutrients. This specificity can make narrow-spectrum toxins more efficient and effective than broad-spectrum ones. Another potential advantage of narrow-spectrum toxins is that a producer can avoid harming other species upon which it depends, which may occur, albeit in a minority of cases (38, 39). While we do not explicitly model this effect here, the expectation is that this would only further favor narrow-spectrum toxins.

The advantage of a narrow-spectrum toxin is seen in the dynamics of competition within a given patch between a broad- and narrow-spectrum toxin user (Fig. 3). By avoiding the wider community, the narrow-spectrum toxin is degraded much less and can accumulate to much higher levels to inhibit conspecific strains, as compared to a broad-spectrum toxin. We note that this advantage to narrow-spectrum toxins would be lost if they were to bind to nontarget cells. However, the biochemistry of narrow-spectrum toxins is such that they are expected to bind more strongly to the strains that they inhibit than to those that they do not. For example, many bacteriocins bind to specific protein receptors that have a restricted phylogenetic range, where the absence of the receptor results in no binding and allows the bacteriocin to continue diffusing (27).

Exploring the model across a wide range of parameter space, including changes in nutrient abundance (*N*), niche overlap (*Ω*), toxin killing effectiveness (*E*), growth rate (*r*), toxin absorption (*θ*), and small changes in arrival time to a patch, all yield narrow-spectrum toxins as the evolutionary stable strategy (*Methods* and *SI Appendix*, Figs. S2 and S3). We also consider an alternative version of the model where the cells of a toxin producer also contribute to the loss of its own toxin (“soaking”) (40), which again gives the same prediction (*SI Appendix*, Fig. S4).

### Broad-Spectrum Toxins Are Most Useful When a Producer Is Abundant.

Our model reveals the potential for a major evolutionary advantage to narrow-spectrum toxins. Whenever toxins are removed from a system as a by-product of their interaction with target cells, being narrow-spectrum can ensure that a toxin is used where it is most needed, that is, against the strongest competitor of a focal strain. As just discussed, there are good reasons to believe that this form of toxin removal commonly occurs, as any toxin that binds or enters a target cell is expected to be made less available for killing through these effects (13, 27, 34, 35, 41). For example, a recent study found that the binding of bacteriocins to *E. coli* target cells creates a major toxin sink because, while it only takes a few toxin molecules to kill a cell, each cell can carry hundreds of receptors that bind the toxins (27). Our prediction that narrow-spectrum toxins can have such a strong advantage is also broadly borne out by their frequent use by bacteria, in particular, the prevalence of bacteriocins (9). However, bacteria also use broad-spectrum toxins, including canonical antibiotics, which raises the question of why these have evolved. To explore this, we decided to expand our model for a wider range of conditions to determine whether we ever find that broad-spectrum toxins are predicted to evolve.

**Fig. 4.**
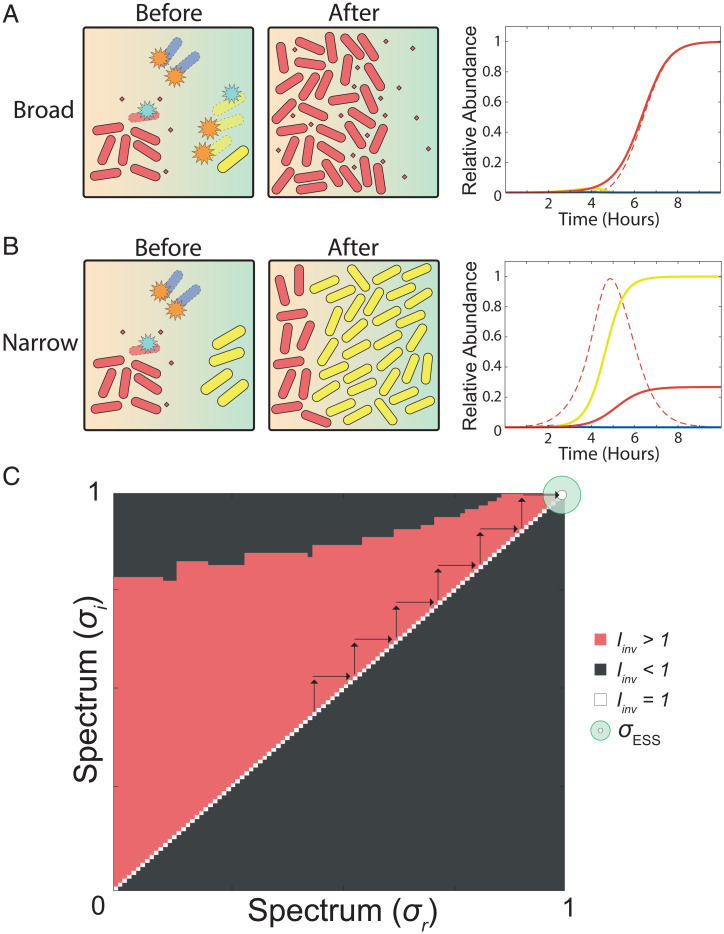
Broad-spectrum toxins evolve when strains are locally abundant. In this model, we ask what happens when strains arrive at different times, such that the focal strain will sometimes arrive first, and sometimes last. Cases where the focal strain arrives first drive its evolutionary dynamics, as this is when the great majority of cells are made. For this reason, we only show this case in *A* and *B*, but all cases are included in the calculation of the ESS. (*A*) The focal strain (red) arrives first, produces broad-spectrum toxins, and completely dominates the environment. Representative temporal dynamics are shown on the right for initial relative abundances of *A* = 1; *B* = 0.4; *C* = 0.7, and other parameters *σ_A_* = 1.0; *σ_B_* = 1.0; *γ_A_* = *γ_B_* = *γ_ESS_* (0.095638). (*B*) The focal strain arrives first and produces a narrow-spectrum toxin, which does not inhibit the community, resulting in limited population growth. Representative temporal dynamics are shown on the right for initial relative abundances of *A* = 1; *B* = 0.4; *C* = 0.7, and other parameters *σ_A_* = 0; *σ_B_* = 0; *γ_A_* = *γ_B_* = *γ_ESS_* (0.035067). For these illustrative plots, single-species abundances are normalized to a maximum abundance of 1.0, and combined toxin abundances are normalized to a maximum of 1.0, which allows the different dynamics to be easily seen. (*C*) PIP for spectrum of activity (*σ*) with density-dependent toxin degradation and variation in arrival time. These conditions lead to an evolutionary stable spectrum strategy that is indiscriminately broad (*σ_ESS_* = 1). For this plot, initial abundances are early arrival: 1.0; late arrival: 0.4; and average arrival: 0.7. All parameters are default unless otherwise indicated.

This analysis revealed that the relative abundance of the focal strain is critical to whether broad- or narrow-spectrum toxins are predicted to evolve. An important cause of variability in relative abundance in natural systems is order of arrival (42), where early-arriving strains can benefit from an early population expansion and a resulting higher abundance than late-arriving strains. We can explore the impact of this effect on evolution in the focal species by assuming that there are now two possible scenarios for within-species competition: Half of the time, the focal strain arrives before the conspecific strain, and, in the other half, the reverse is true (Fig. 4). In other words, this model incorporates variation in starting frequency. In each case, we assume, for simplicity, that the rest of the community arrives at the average of the arrival time of the two strains, that is, halfway between their respective arrival times. By systematically increasing the disparity in arrival time, we identified a point where there is a shift toward natural selection for broad-spectrum toxins (Fig. 4*C* and *SI Appendix*, Fig. S3).

What drives this evolution of broad-spectrum toxins? The great majority of natural selection on the focal strain occurs in the cases where it arrives early, because this is when it reaches a high abundance and generates the vast majority of its cells across patches. Here, the focal strain is ecologically dominant, outnumbering both its conspecific competitor and the community. This abundance means the focal strain can produce enough of a broad-spectrum toxin to overcome the high levels of toxin loss and, thereby, inhibit all species in the patch. The result is complete dominance of a local patch (Fig. 4*A*). As such, making a narrow-spectrum toxin under these conditions results in a lost opportunity for the early-arriving strain to remove all competitors from the patch.

To further investigate this effect, we can also vary the arrival time of the community. As expected, when the community arrives earlier, this can undermine the benefit of using a broad-spectrum toxin, and again lead to natural selection for narrow-spectrum toxins. Early arrival by the wider community results in the broad-spectrum toxin being lost to targeting these cells and no longer accumulates or inhibits effectively (*SI Appendix*, Fig. S5). Again, this predicts that broad-spectrum toxin evolution is associated with conditions where a strain is locally abundant and ecologically dominant.

Finally, we consider the possibility that a focal strain is always at high initial abundance (rather than only some of the time due to arriving early). This could occur, for example, if the strain is an intrinsically better disperser than all of the other bacterial strains in the patch and so it reliably arrives earlier. An important implication of this scenario is that the ecology of the focal strain is now distinct from all other bacteria in the patch, including the niche competitor that we previously assumed was a conspecific. We therefore no longer assume that evolutionary changes in the focal strain are also reflected in its key competitor strain; that is, they are no longer conspecifics in the same gene pool. Put another way, we now treat these two strains as different species that are no longer coevolving. To study toxin evolution in this scenario, we then ask, simply, what is the best strategy for the focal species to maximize its abundance? In agreement with our earlier coevolutionary models (Fig. 4), we observe the evolution of broad-spectrum toxins whenever the focal strain starts at considerably greater abundance than its key niche competitor (*SI Appendix*, Fig. S6). Moreover, as we increase the starting abundance of the community, natural selection for broad-spectrum toxins requires a corresponding increase in focal strain abundance (*SI Appendix*, Fig. S7). Despite the differing assumptions from our main model, therefore, this final analysis further supports the prediction that the relative abundance of a producer is critical for the evolution of broad-spectrum toxins.

### A Meta-analysis Suggests That Broad-Spectrum Toxins Are Deployed at High Cell Abundances.

Our model predicts narrow-spectrum toxins often carry a major advantage, but broad-spectrum toxins can become effective when a strain is abundant enough to compete effectively with multiple species. This density dependence for broad-spectrum toxins is consistent with the general prediction from recent mathematical models and experiments that show that toxin efficacy can depend strongly on producer abundance (33). However, there are other factors that may select for broad-spectrum toxins that are not captured by our model. For example, ecologies with highly unpredictable competitor species could favour broad-spectrum toxins that are more able to inhibit diverse competitor species than narrow-spectrum ones. Therefore, we sought an experimental test of our prediction that strain abundance is linked to the benefits of using broad-spectrum toxins. There are few data on the relative abundance of microbial species at the fine spatial scales at which they interact and compete with one another (27, 43), and even if such data were available, we are unlikely to know the toxins and their spectrum for most species. However, if our predictions are correct, we reasoned that one should be able to observe a difference in the regulation of narrow- and broad-spectrum toxins. Specifically, our modeling predicts that broad-spectrum toxins should mostly be used when a producer is at high abundance.

In order to explore the potential relationship between the spectrum of a toxin and its regulation, we compiled a dataset of antibacterial toxins with the goal of identifying all well-understood examples that have information on the toxins’ spectrum of activity, regulation, and molecular targets. We include a known molecular target to help select well-understood examples with high-quality data. Target information also helps to validate experimental data on spectrum, based upon how widespread the target is across strains and species. From the data, we identify 71 well-studied toxins for which our conditions for inclusion are satisfied (*Methods*). For each of these, we classify the nature of each toxin’s form of regulation—in particular, whether their expression is likely to be up-regulated at high density—and their spectrum. Further details of mechanisms of regulation, spectrum, and receptors are available in *SI Appendix*, Table S1, which also provides references for the categorizations of all toxins included in our analysis.

For regulation, we use three categories to broadly capture the observed variation. We group regulatory networks that are most likely to drive density-dependent responses under one category. Central among these is quorum sensing, whereby cells release a small molecule from the cell (44). By monitoring the concentration of this small molecule as it builds up, cells can infer the density of bacteria around them producing this molecule. Also included as density-dependent regulation mechanisms are those related to sporulation (e.g., Spo0A) (45) and biofilm conditions (OmpR) (30, 46), whose expression have both been linked to high density. A second key category is stress responses that specifically respond to nutrient limitation, such as iron deprivation, nitrogen starvation, and the stringent response. Nutrient limitation may sometimes be indicative of high cell density, but it is also likely that many bacteria occur at low density because they are in low-nutrient conditions, giving the opposite pattern. We, therefore, do not consider nutrient limitation a reliable indicator of high cell density. The other major category is that of DNA damage–mediated regulation, specifically, the SOS response, which is known to regulate many well-characterized toxins including many colicins and pyocins (6, 47). DNA damage–mediated regulation is not considered to be an indicator of high cell density.

We categorize the spectrum of toxins based upon the phylogenetic diversity of the species that can be inhibited. This approach allows us to be systematic and avoid the subjectivity that can come with comparing one particular toxin, or group of toxins, to others (48). Even toxins that are considered narrow-spectrum, such as the canonical narrow-spectrum colicins of *E. coli*, can inhibit bacteria from different families (49). We therefore use the within one class level for the narrow-spectrum category (spectrum value of 1), and then within one phylum (spectrum value of 2) and targets multiple phyla (spectrum value of 3) for increasingly broad-spectrum toxins. A final caveat is that, just because a toxin can be shown to inhibit diverse species, this does not necessarily mean that it will in nature, where these diverse species may not be present. Nevertheless, these experimental measures of toxin spectrum are likely to be a good general indicator of their activities in natural contexts.

**Fig. 5.**
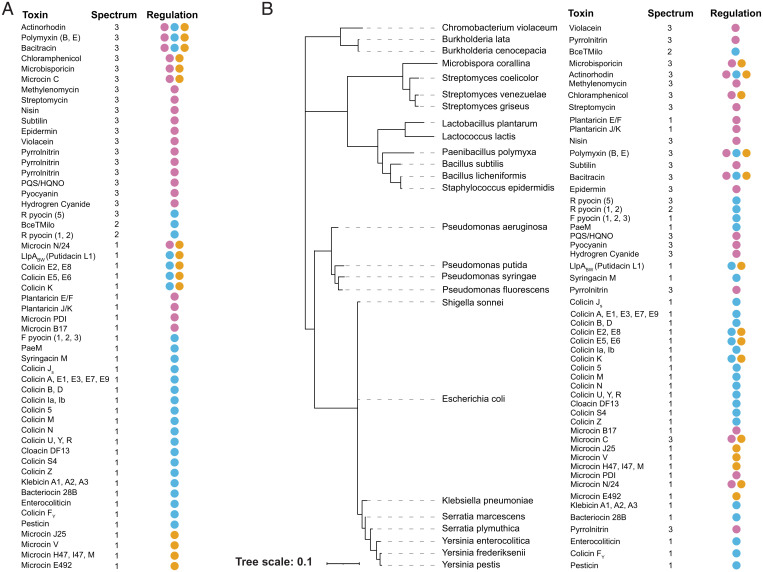
Broad-spectrum toxins are associated with density-dependent regulation. (*A*) Ranking antibacterial toxins by spectrum of activity indicates a strong association with density-dependent regulation. Pink: density-dependent; blue: SOS response; orange: nutrient stress. Spectrum of activity based on experimentally demonstrated susceptibility is as follows: 1, within one class; 2, within one phylum; 3, across multiple phyla. (*B*) The association between spectrum and regulation remains when one controls for phylogenetic effects (see main text for statistics). Here we show the 16S ribosomal RNA based phylogeny of the toxin producing species in our analysis with toxin, regulation, and toxin spectrum of activity metadata. Toxins that share regulation, spectrum, and target are listed as a single line entry. Further details and references are provided in *SI Appendix*, Table S1.

Simply ranking the toxins by their spectrum immediately indicates a strong association with regulation mode (Fig. 5*A*), where density-dependent regulation is strongly enriched within the broad-spectrum toxins. Specifically, the toxins we categorized as being under density-dependent regulation, based on the literature, have a mean spectrum value of 2.71 and a median value of 3 (the maximum of our measure of spectrum, previous paragraph). This contrasts strongly with the non-density-dependent regulation toxins, which have a mean spectrum value of 1.14 and a median value of 1 (the minimum of our measure of spectrum). However, a caveat is that it is possible for such associations to occur due to phylogenetic biases. For example, if there is a large clade of bacteria that all have density-dependent regulation and broad-spectrum toxins, this could drive the association, when, in fact, it only evolved on one occasion in one common ancestor.

We can account for any such effects by putting the species on a phylogeny (Fig. 5*B*) and then testing the effect of spectrum on the mode of regulation using a statistical model that incorporates phylogenetic effects [binaryPGLMM (50, 51)]. More specifically, we assess the likelihood of toxins with spectrum value 2 (intermediate) or 3 (broad) being regulated by a density-dependent mechanism (classified as a binary presence or absence variable) compared to toxins with a spectrum value of 1 (narrow) by fitting a logistic model. There is no effect when comparing narrow (spectrum value of 1) and intermediate (spectrum value of 2) toxins (*SI Appendix*, Table S2), which is expected as there are few examples of intermediate toxins. However, comparing narrow (spectrum value of 1) and broad (spectrum value of 3), we find, as predicted, a highly significant association between broad-spectrum toxins and density-dependent regulation (*β*: 4.1699; S.E.: 1.2196; *P <* 0.001). Putting the statistical results into more relatable terms, *β* is the log-odds ratio between our two groups of interest. By exponentiating *β*, this gives that broad-spectrum toxins are ∼60 times more likely to be under density-dependent regulation than narrow-spectrum toxins. This analysis, therefore, supports a strong interaction between density-dependent regulation and the use of broad-spectrum toxins. To ensure these results were not dependent upon our statistical method, we used another widely used statistical model, MCMCglmm (52), which is a Bayesian statistical model that accounts for phylogeny as a random effect to account for dependence in the data. This model again supported a strong effect between density-dependent regulation and broad-spectrum toxins (*SI Appendix*, Table S2, “Main analysis”).

One potential criticism of our analysis is that we have included some forms of regulation—regulators of sporulation and biofilm formation—that are not always thought of as density-dependent regulation to the same extent as quorum sensing. We believe the inclusion of these additional forms of regulation as density dependent makes sense given the available data (46, 53). Nevertheless, we decided to test whether the inclusion of these additional regulators was critical for our findings. To do this, we took a more stringent definition of density-dependent regulation that only includes canonical quorum sensing. We again see the same patterns. For density-dependent toxins, we find a mean spectrum value of 2.71 and a median value of 3, while those not regulated by density-dependent mechanisms have a mean spectrum value of 1.33 and a median value of 1. The phylogenetic analysis is again significant for both statistical methods (binaryPGLMM spectrum 1 vs. 3; *β*: 2.9727; S.E.: 0.9944; *P* value < 0.005; for Bayesian test, see *SI Appendix*, Table S2, “Strict Analysis”**)**. While we favor our initial definition of density-dependent regulation, therefore, we still find strong evidence for a link between density dependence and the use of broad-spectrum toxins when we apply a more stringent definition.

### Conclusions.

Spectrum of activity is a key property of any antimicrobial compound, and yet the evolutionary origins of this property have received little attention. Here, we have identified an evolutionary rationale for the evolution of both narrow- and broad-spectrum toxins during bacterial warfare. Our models predict that narrow-spectrum toxins will evolve to target competing strains that are the greatest threat, without wasting toxin investment on other species which are less of a threat (Fig. 3). However, when a strain is sufficiently abundant, natural selection can, instead, favor broad-spectrum toxins that allow a strain to cement its ecological dominance by suppressing not only their key competitors but also members of the wider community (Fig. 4).

We tested this prediction with a phylogenetically controlled meta-analysis of published data, which identified a strong link between density-dependent regulation and the use of broad-spectrum toxins (Fig. 5). This suggested, as predicted, that bacteria have evolved to use broad-spectrum toxins when they are at relatively high abundance. It is interesting to consider alternative explanations for this link between broad-spectrum toxins and density-dependent regulation. For example, if broad-spectrum toxins are inherently less potent, and therefore require higher concentrations to be effective, this could favor density-dependent regulation. However, the data suggest that broad-spectrum toxins can be both highly effective and under density-dependent regulation [e.g., colistin and nisin are inhibitory at comparable concentrations to the most potent of the narrow-spectrum colicin bacteriocins (54–57)]. While changes in efficacy may be a contributing factor, therefore, it does not appear to explain the data. More generally, our meta-analysis only considered bacteria, where there is the most information on regulation and spectrum of antimicrobials. However, our predictions are generally applicable to other microbes. In fungi, for example, we might expect similar links between abundance and the benefits of broad-spectrum toxins.

There is an ongoing discussion of the relative merits of broad- vs. narrow-spectrum antimicrobials as a treatment strategy (8, 32, 58). Traditionally, broad-spectrum antibiotics have been favored, as they enable treatment of a wide range of conditions without specific knowledge of the causal agent. However, narrow-spectrum alternatives, including the bacteriocins, are increasingly being considered due to their ability to target a given pathogen without negatively affecting the commensal community (8, 32, 58, 59). Our evolutionary modeling suggests another benefit to their use: By targeting a specific species, less of a given drug may be lost to off-target effects. This targeting has the potential to improve delivery, particularly in dense communities where large amounts of a given drug can be degraded or absorbed by targeted cells (27, 34, 60). More generally, our model underlines the great potential for bacteria and other microbes to evolve narrow-spectrum toxins. In line with this prediction, there is a growing number of bacteriocins being discovered (61–64), which raises the possibility of a large, and largely untapped, diversity in these antimicrobial compounds. Another prediction of our modeling is that a focus on abundant species may inadvertently enrich for antibiotic production, because it is ecologically dominant species that stand to benefit the most from broad-spectrum toxins. If the goal is to shift to narrow-spectrum toxins, therefore, there may be value in a focus on the less abundant taxa.

## Methods

Our methods closely follow published work on the evolution of competition in bacteria (10, 11, 18). These methods combine 1) a realistic description of the dynamics of bacterial population growth within competitions with 2) approaches from evolutionary game theory that take the outcomes of individual competitions and use them to predict evolutionary outcomes. More details of these approaches can be found in the earlier work (10, 11, 18), but we also go through the methods here, with particular focus on the aspects tailored to studying spectrum evolution, which is the key novelty in our work.

### Differential Equations and Default Parameters.

The core of the model is a system of ordinary differential equations (ODEs), which is based on earlier models (11, 13, 17). In the first model, we use a constant term for toxin loss from the system, as is typical (10–12, 14, 17), but we later modify this. We use the model to follow the dynamics of three species, the two toxins made by species *A* and *B*, and two nutrients,

[1] $$\frac{dA}{dt}=A\left(t\right)*\left(\frac{N_{1}(t)}{\left(N_{1}(t)+K_{N1}\right)}*r_{A}*\left(1-\gamma_{A}\right)-E*\left(\frac{T_{B}^{h}(t)}{T_{B}^{h}(t)+K_{\;BA}^{h}}\right)\right)$$

[2] $$\frac{dB}{dt}=B\left(t\right)*\left(\frac{N_{1}(t)}{\left(N_{1}(t)+K_{N1}\right)}*r_{B}*\left(1-\gamma_{B}\right)-E*\left(\frac{T_{A}^{h}(t)}{T_{A}^{h}(t)+K_{\;AB}^{h}}\right)\right)$$

[3] $$\frac{dC}{dt}=C\left(t\right)*\left(\frac{N_{2}\left(t\right)}{\left(N_{2}(t)+K_{N2}\right)}*r_{C}+\frac{N_{1}\left(t\right)}{\left(N_{1}(t)+K_{N1}\right)}*\Omega_{1}*r_{C}-E*\left(\frac{T_{A}^{h}\left(t\right)}{T_{A}^{h}\left(t\right)+K_{\;AC}^{h}}\right)-E*\left(\frac{T_{B}^{h}\left(t\right)}{T_{B}^{h}\left(t\right)+K_{\;BC}^{h}}\right)\right)$$

[4] $$\frac{dT_{A}}{dt}=\gamma_{A}*A\left(t\right)*\left(\frac{N_{1}(t)}{N_{1}(t)+K_{N1}}\right)-L_{T}*T_{A}(t)$$

[5] $$\frac{dT_{B}}{dt}=\gamma_{B}*B\left(t\right)*\left(\frac{N_{1}(t)}{N_{1}(t)+K_{N1}}\right)-L_{T}*T_{B}(t)$$

[6] $$\frac{dN_{1}}{dt}=-\left(\frac{N_{1}(t)}{N_{1}(t)+K_{N1}}\right)*(A\left(t\right)+B\left(t\right)+\Omega_{1}*C\left(t\right))$$

[7] $$\frac{dN_{2}}{dt}=-\left(\frac{N_{2}(t)}{N_{2}(t)+K_{N2}}\right)*C\left(t\right),$$

where *A*, *B*, and *C* are the biomass of the focal species, the conspecific (or niche competitor, in the case of *SI Appendix*, Figs. S6 and S7), and the community, respectively. The community, therefore, is modeled as a single species, for simplicity. Extending the model of the community to define multiple individual species (with differing degrees of niche overlap with the focal strain) could result in natural selection for intermediate spectrum toxins, where certain community members are targeted while others are avoided. However, we do not explore this further here. *T_A_* and *T_B_* are the biomass of the focal species toxin and the conspecific (or niche competitor, in *SI Appendix*, Figs. S6 and S7) toxin, respectively. *N*_1_ and *N*_2_ denote nutrient 1 and nutrient 2. As discussed in the *Results and Discussion*, we represent spectrum in terms of toxin-receptor affinity. A small *K_ij_* value results in high affinity of toxin from species *i* to the receptor of species *j*, whereas a large *K_ij_* value results in low affinity of toxin from species *i* to the receptor of species *j*. The affinity of toxins for the receptor of their conspecific are fixed at the highest level of affinity within our model (*K*_min_ = 0.05). This leaves *K_iC_*, the affinity of toxin from species *i* to the receptor of the community (*C*), as the evolving parameter of the spectrum (*σ*) in our model. We convert *K_iC_* to *σ* using Eq. **8** to create a term which varies from zero to one and is more easily interpreted than *K_iC_*. A full list of variables and default parameters can be found in *SI Appendix*, Table S3.

[8] $$\sigma_{i}=1-\left(\frac{K_{iC}-K_{\min}}{K_{\max}-K_{\min}}\right).$$

We modify this model to incorporate a density-dependent toxin loss term. While the majority of previous models take the form of our first model, there are some that have density-dependent toxin loss, and we follow these in this second model (13, 16, 33). This modification means the model now captures the potential for target cells to drive loss of the toxin from the system, such as would occur if they degrade or bind the toxin. We also include a toxin absorption term (θ) with a default value of one, yet modify this term as part of our parameter sweeps. This second model replaces Eqs. **4** and **5** with Eqs. **9** and **10**.

[9] $$\frac{dT_{A}}{dt}=\gamma_{A}*A\left(t\right)*\left(\frac{N_{1}\left(t\right)}{N_{1}\left(t\right)+K_{N1}}\right)-\theta *\left(B\left(t\right)*\left(\frac{T_{A}^{h}\left(t\right)}{T_{A}^{h}\left(t\right)+K_{\;AB}^{h}}\right)+C\left(t\right)*\left(\frac{T_{A}^{h}\left(t\right)}{T_{A}^{h}\left(t\right)+K_{\;AC}^{h}}\right)\right)$$

[10] $$\frac{dT_{B}}{dt}=\gamma_{B}*B\left(t\right)*\left(\frac{N_{1}\left(t\right)}{N_{1}\left(t\right)+K_{N1}}\right)-\theta *\left(A\left(t\right)*\left(\frac{T_{B}^{h}\left(t\right)}{T_{B}^{h}\left(t\right)+K_{\;BA}^{h}}\right)+C\left(t\right)*\left(\frac{T_{B}^{h}\left(t\right)}{T_{B}^{h}\left(t\right)+K_{\;BC}^{h}}\right)\right).$$

We use these ODEs to model outcomes of toxin-based bacterial competition within a given patch (Figs. 1*B*, 2 *A* and *B*, , and and *SI Appendix*, Fig. S5 *A* and *B*), and we then use game theory to predict, from these competitions, the evolution of both toxin production rate and spectrum, as discussed in the next section. For one analysis (*SI Appendix*, Fig. S4), we also include a term for toxin loss driven by the producer strain, replacing Eqs. **9** and **10** with Eqs. **11** and **12**. This term is set to the strongest toxin-receptor affinity.

[11] $$\frac{dT_{A}}{dt}=\gamma_{A}*A\left(t\right)*\left(\frac{N_{1}\left(t\right)}{N_{1}\left(t\right)+K_{N1}}\right)-\theta *\left(A\left(t\right)*\left(\frac{T_{A}^{h}\left(t\right)}{T_{A}^{h}\left(t\right)+K_{\;AA}^{h}}\right)+B\left(t\right)*\left(\frac{T_{A}^{h}\left(t\right)}{T_{A}^{h}\left(t\right)+K_{\;AB}^{h}}\right)+C\left(t\right)*\left(\frac{T_{A}^{h}\left(t\right)}{T_{A}^{h}\left(t\right)+K_{\;AC}^{h}}\right)\right)$$

[12] $$\frac{dT_{B}}{dt}=\gamma_{B}*B\left(t\right)*\left(\frac{N_{1}\left(t\right)}{N_{1}\left(t\right)+K_{N1}}\right)-\theta *\left(A\left(t\right)*\left(\frac{T_{B}^{h}\left(t\right)}{T_{B}^{h}\left(t\right)+K_{\;BA}^{h}}\right)+B\left(t\right)*\left(\frac{T_{B}^{h}\left(t\right)}{T_{B}^{h}\left(t\right)+K_{\;BB}^{h}}\right)+C\left(t\right)*\left(\frac{T_{B}^{h}\left(t\right)}{T_{B}^{h}\left(t\right)+K_{\;BC}^{h}}\right)\right).$$

Default parameters are used throughout (*SI Appendix*, Table S3), except where noted. All equations are allowed to proceed until steady state, as defined by the point where the change in nutrient abundance is less than 5e^−8^ ($\text{Max}(|dN_{1}/dt|,\;|dN_{2}/dt|)\;\;<{\text{5e}}^{-8}$), which proved a reliable proxy for steady state across all variables. Once at steady state, abundance of the focal strain is measured.

### Game Theory.

The differential equation models predict the outcome of a given competition between our focal strain, its conspecific competitor, and the wider community. We embed these competitions in a broader framework of game theory, known as adaptive dynamics (20), which we use to understand how traits evolve over successive rounds of competition. Unlike some models which focus on a single trait, we follow the evolution of two traits, toxin production rate and toxin spectrum, in two steps.

#### Step 1: Evolution of production rate *γ*_ESS_.

We perform multiparameter optimization with a two-step process where we first ask, what is the evolutionarily stable production rate—the ESS (65)—(*γ_ESS_*), under the assumption that both strains are producing toxins with equal spectrum (*σ_A_* = *σ_B_*)? In this first step, we make no assumptions about the optimum spectrum strategy, but rather, we ask, what is the evolutionarily stable production rate (*γ_ESS_*) for every possible spectrum strategy (*σ*) from zero to one?

To establish an evolutionary stable strategy (in this case, *γ_ESS_*), we employ a form of adaptive dynamics (11, 66, 67) (*SI Appendix*, Fig. S1). We consider a resident bacterial strategy of *γ*_res_, which gives the current toxin production rate in the focal species. Following previous work, we assume that the bacteria live in a patchy landscape, which could be a series of hosts, where each competition modeled by the differential equations occurs at the scale of one patch. Any competition in one patch, therefore, is assumed to be one of a large number that can occur in parallel. In this population, the fitness of the resident strategy (*ω*_res_) is assessed from the number of cells it makes when the focal strain uses its *γ*_res_ strategy in competition with its conspecific strain using the identical *γ*_res_ strategy. We do not model the evolution of toxin diversity here, but it is commonly high in natural populations (9, 21, 22). Therefore, again following previous work (11, 23), we assume that toxin diversity is high, such that any two strains that meet will have toxins that they are able to use against each other. Next, we calculate the fitness of a new (invading) strategy (*ω*_inv_). Because this strategy is rare, it is not expected to meet the same strategy in a patch. Instead, it will meet the resident strategy. Accordingly, the fitness of the invading strain is taken from the number of cells it produces when the focal strain uses *γ*_inv_ strategy, while in competition with its conspecific strain using the *γ*_res_ strategy. We then calculate the invasion index (*I*_inv_) for the invading strategy as previously (11, 66, 67), where

[13] $$I_{\text{inv}}=\frac{\omega_{\text{inv}}}{\omega_{\text{res}}}=\frac{\omega \left(\gamma_{\text{inv}}\;|\;\gamma_{\text{res}}\right)}{\omega \left(\gamma_{\text{res}}\;|\;\gamma_{\text{res}}\right)}.$$

We calculate the invasion index for all possible pairs of resident and invader strategies, and visualize the results as a pairwise invasibility plot (PIP) (20) (*SI Appendix*, Fig. S1). When *I*_inv_ is greater than one (red regions in *SI Appendix*, Fig. S1), the invading strategy can invade, and, when *I*_inv_ is less than one (black regions in *SI Appendix*, Fig. S1), the invading strategy cannot invade and is lost. By definition, when the invading strain and the resident strain have the same value, they will achieve equal biomass and *I*_inv_ = 1 (shown in white in our PIPs). A *γ_ESS_* in a PIP is the point on the diagonal where no other strategy can invade, representing a new monomorphic metapopulation utilizing the *γ_ESS_* strategy. Visually, for any given PIP, the ESS will appear as a white point along the diagonal where there is no red above or below it. For most of the PIPs for the spectrum in this work, the ESS is at the edge of the bounded interval, and so the white point on the diagonal is bounded by the outer edge of the PIP, and a column of black above or below.

#### Step 2: Evolution of spectrum σESS.

In the second step, we equip both the focal strain and the conspecific with the evolutionarily stable production rate (*γ_ESS_*) for each value for the spectrum (*σ*), as determined in the first step. We again use adaptive dynamics and invasion analysis, except now we measure the *I*_inv_ based on variability in the spectrum (*σ*) strategy. Specifically, we calculate the fitness of the resident strategy (*ω*_res_) by measuring the final biomass achieved when the focal strain uses *σ*_res_ and the corresponding *γ_ESS_*, while in competition with its conspecific strain using the identical *σ_res_* strategy and *γ_ESS_*. We then calculate the fitness (*ω*_inv_) of a new mutant *σ* strategy (*σ*_inv_) (using the same *γ*_ESS_) by measuring the biomass of the mutant focal species when in competition with the conspecific strain utilizing the *σ*_res_ and *γ_ESS_* strategy. Again, assuming that the production rate can rapidly evolve (9, 11, 29, 30), with each successful invasion of a new spectrum strategy, the production rate automatically shifts to the *γ_ESS_* for the new *σ*_res_. We then repeat the invasion analysis as before, establishing the evolutionary stable state of *σ* (*σ_ESS_*) (Figs. 2–4 and *SI Appendix*, Figs. S2–S5).

[14] $$I_{\text{inv}}=\frac{\omega_{\text{inv}}}{\omega_{\text{res}}}=\frac{\omega \left(\sigma_{\text{inv}}\;|\;\sigma_{\text{res}}\;\right)}{\omega \left(\sigma_{\text{res}}\;|\;\sigma_{\text{res}}\right)}.$$

It is typical, in adaptive dynamics, to focus on small changes in trait values in a mutant relative to a resident when making predictions. Our models perform well under this assumption, but, in most cases, they also perform well for large changes in trait value, owing to the simplicity of the resulting PIPs. For example, Figs. 2–4 would ultimately be expected to reach the same ESS with large steps sizes, so long as there are also occasional small step sizes to allow optimization.

### Modeling Variability in Arrival Time.

For the competitions using asymmetric starting ratios (Fig. 4 and *SI Appendix*, Figs. S3 and S5), the fitness of a given strategy was calculated from the average number of cells produced across patches when the focal strain arrives early, and then when the conspecific arrives early. For example, the results shown in Fig. 4 use starting values of 1e^−4^ for the early-arriving strain (default starting abundance), and 0.4e^−4^ for the late-arriving strains, which is a simplified way to model what would occur if the early-arriving strain had the chance to undergo population growth before the late-arriving strain appeared. We again use the two-step process as described above (*SI Appendix*, Fig. S1) to determine the optimized production rate (*γ_ESS_*) for each value of the spectrum (*σ*), using the average biomass produced for a given strategy (when both arriving early and arriving late) when calculating the fitness (ω) for a given strategy. We repeat this same process again to calculate the optimum spectrum (*σ_ESS_*). For *SI Appendix*, Fig. S5, we use the exact same methodology, except now the community species is already established in each case (starting abundance 3.0e^−4^).

It could be argued that variation in starting abundance is too simplifying of an assumption for modeling variation in arrival time. Therefore, we also develop a model which allows the early-arriving strain to grow and produce toxins prior to the arrival of other competitors, although this results in a more complex numerical model (*SI Appendix*, Fig. S3*B*). We achieve this by allowing the early-arriving strain to increase its own abundance by a specified percentage in the absence of the community and the conspecific competitor. The community always arrives halfway between the arrival of the two strains of the focal species. This requires stopping and restarting the numerical solver for the addition of each new strain. Each strain then arrives with the same starting abundance (1e^−4^). This more complex version of the model does not qualitatively affect predictions, so we focus on the simpler version.

### Toxin Spectrum, Regulation, and Target Receptor Survey.

We began compiling a list of bacterial toxins for our meta-analysis by downloading all current entries, as of March 17, 2021, in the BAGEL4 database (1,005 entries), which is a database of ribosomally synthesized peptide/protein bacterial toxins. Each entry in BAGEL4 was individually examined to determine whether experimental data were available regarding regulation, spectrum of activity, and target receptor. The first reference examined in each case was the reference associated with the BAGEL4 entry. If this reference lacked the necessary information, all reasonable effort was made to find supplementary references to provide the relevant data for inclusion in this analysis. The spectrum of activity data required susceptibility testing against at least one species other than the producing species. This requirement only excluded S pyocins (S1, S2, S3, and S4) of *Pseudomonas aeruginosa*. Even so, there remains the potential for our estimates of spectrum to be influenced by how widely a given toxin was tested. Toxins in the BAGEL4 database that did not include a reference or any clear identifying information (e.g., Class I lanthipeptide 001) were excluded from consideration. A single toxin, lacticin Q produced by *Lactococcus lactis* strain QU5, met our requirements for inclusion, yet was excluded from Fig. 5 and the corresponding meta-analysis. The positive regulator of lacticin Q production, LnqR, is attenuated at elevated temperatures, leading to decreased lacticin Q production (68). This form of regulation does not fit naturally with any of the typical categories, and, given it is a one-off, we deemed it unhelpful to include it.

As discussed above, the BAGEL4 database exclusively contains ribosomally synthesized peptides/proteins, which excludes many other molecules which are often implicated as interbacterial toxins. No similar database exists to catalog such compounds. We therefore compiled a list manually for the other classes of toxins, which include small-molecule antibiotics, with the goal, again, of finding all toxins that meet our criteria. All toxins included in the analysis, and relevant citations, are included in *SI Appendix*, Table S1.

### Supplementary Methods.

For additional methods regarding the statistical meta-analysis and default parameter values for the models, please see *SI Appendix*. All code and data from this work can be found at doi:10.5281/zenodo.6819262.

## Supplementary Material

Supplementary File

## Data Availability

Matlab scripts, R scripts, and data files have been deposited in GitHub: JPox14/PNAS_2022-05407 (69). All code and data from this work can be found at doi: 10.5281/zenodo.6819262 (70).
