# Supplementary material for: The evolution of spectrum in antibiotics and bacteriocins
Source: Proc Natl Acad Sci U S A. 2022 Sep 13;119(38):e2205407119. doi: 10.1073/pnas.2205407119 (PMC9499554; doi:10.1073/pnas.2205407119)
Supplement: Supplementary File [file pnas.2205407119.sapp.pdf]

PNAS

Supporting Information for
The evolution of spectrum in antibiotics and bacteriocins

Jacob D. Palmer and Kevin R. Foster

Jacob D. Palmer
Email: jacob.palmer@biology.ox.ac.uk

Kevin R. Foster
Email: kevin.foster@biology.ox.ac.uk

This PDF file includes:

Supporting text
Figures S1 to S7
Tables S1 to S3
SI References

Supporting Information Text

Methods

Parameter sweeps

We use a core default set of parameters throughout to facilitate comparison between models and results (**Table S3**). However, we performed a number of parameter sweeps to confirm the robustness of our findings to changes in key parameters, including initial pool of nutrients (N), degree of niche overlap (Ω), toxin killing efficiency (E), growth rate (r), toxin absorption (θ), and differences in starting abundances (**Figure S2, Figure S3**).

Throughout, the parameters studied focus on conditions where toxin use is under positive natural selection because, if toxin use is disfavored, the evolution of spectrum becomes irrelevant e.g., when there is a very low toxin killing efficiency (E). One consequence of this is that we need to use a higher default value for toxin-killing efficiency for the second model, which uses cell-density dependent toxin degradation, than for the first model that uses constant degradation. This change is needed to avoid the toxins becoming ineffective in the second model where degradation rates can become high. However, we show that our conclusions hold when toxin-killing efficiency is decreased, or increased, within the range where toxin production is under positive natural selection (**Figure S2d**).

16S rRNA Phylogeny and Statistical Analysis

16S rRNA sequences for each species with a toxin included in the analysis were downloaded from the SILVA database and selected based on maximum sequence quality. Alignment was carried out using MEGA X with default settings, alignment by ClustalW(*1*), with a gap opening penalty of 15 and a gap extension penalty of 6.66. The evolutionary history was inferred by using the Maximum Likelihood method and Tamura-Nei model(*2*). The tree with the highest log likelihood (-9094.78) is shown, using 1500 bootstrap replications. The percentage of trees in which the associated taxa clustered together is shown next to the branches. Initial tree(s) for the heuristic search were obtained automatically by applying Neighbor-Join and BioNJ algorithms to a matrix of pairwise distances estimated using the Tamura-Nei model, and then selecting the topology with

superior log likelihood value. A discrete Gamma distribution was used to model evolutionary rate differences among sites (5 categories (+G, parameter = 0.6027)). The rate variation model allowed for some sites to be evolutionarily invariable ([+I], 27.72% sites). The tree is drawn to scale, with branch lengths measured in the number of substitutions per site. This analysis involved 25 nucleotide sequences. All positions with less than 90% site coverage were eliminated, i.e., fewer than 10% alignment gaps, missing data, and ambiguous bases were allowed at any position (partial deletion option). There were a total of 1324 positions in the final dataset. Evolutionary analyses were conducted in MEGA X(*3*).

For the meta-analysis, we repeat the phylogenetic analysis as described above, but must include repeat 16S reference sequences for each species which has >1 toxin. We exported the phylogeny (phylo) in .nex file format as input for the binaryPGLMM(*4*, *5*) package in R. Spectrum of activity was recorded on a scale of 1 to 3 (Spectr), as demonstrated in **Figure 5**, and regulation was categorized based on the presence or absence of density-dependent regulation (DDR). Model specifications were as follows: binaryPGLMM(formula = DDR~as.factor(Spectr), phy = phylo, data = dater). Model outputs are reported as the log-odds ratio (β) of density-dependent regulation (DDR = 1) for a broad-spectrum toxin (Spectr = 3) or intermediate spectrum toxin (Spectr = 2) compared to a narrow-spectrum toxin (Spectr = 1); as well as standard error (S.E.), z-score and *p*-value. *p*-values < 0.05 considered statistically significant.

To ensure the statistical outcomes are not inappropriately biased by the specific model selected, we subsequently repeated the statistical analysis with the Bayesian MCMCglmm(*6*) package in R using the same phylogeny used to generate **Figure 5b**. We are investigating a correlation between spectrum of activity and density-dependent regulation, and therefore we use spectrum of activity as a fixed effect and the inverse of the phylogeny matrix as a random effect. We use a weakly informative Gelman prior for the fixed effect(*7*, *8*), an inverse Wishart distribution for the random effect, and set residual variance to one. Collectively, we model the Bayesian analysis after ref (*8*). Code for prior and model specification was as follows: prior <- list(B=list(mu=c(0,0), V=gelman.prior(~as.factor(Spectr), data=dader, scale=1+1+pi^2/3)), R=list(V=1, fix=1), G=list(G1=list(V=diag(1)*0.1, nu=1))). model_b<-MCMCglmm(DDR~as.factor(Spectr),

random=~Bacteria, family="categorical", ginverse=list(Bacteria=inv.filo1$Ainv), verbose=FALSE, prior=prior, data=dader, scale = F, slice = T, nitt = 1000000, burnin = 5000, thin = 50). Model outputs are reported as the posterior distribution mean (μ), which is the log-odds ratio of density-dependent regulation (DDR = 1) for a broad-spectrum toxin (Spectr = 3) or intermediate spectrum toxin (Spectr = 2) compared to a narrow-spectrum toxin (Spectr = 1); 95% credible intervals (CI), and *pMCMC*-values, each of which are the average of three individual runs of the model.

We use both binaryPGLMM and MCMCglmm for our meta-analysis, as has been done previously(*9*). Full statistical results are provided in **Table S2**.

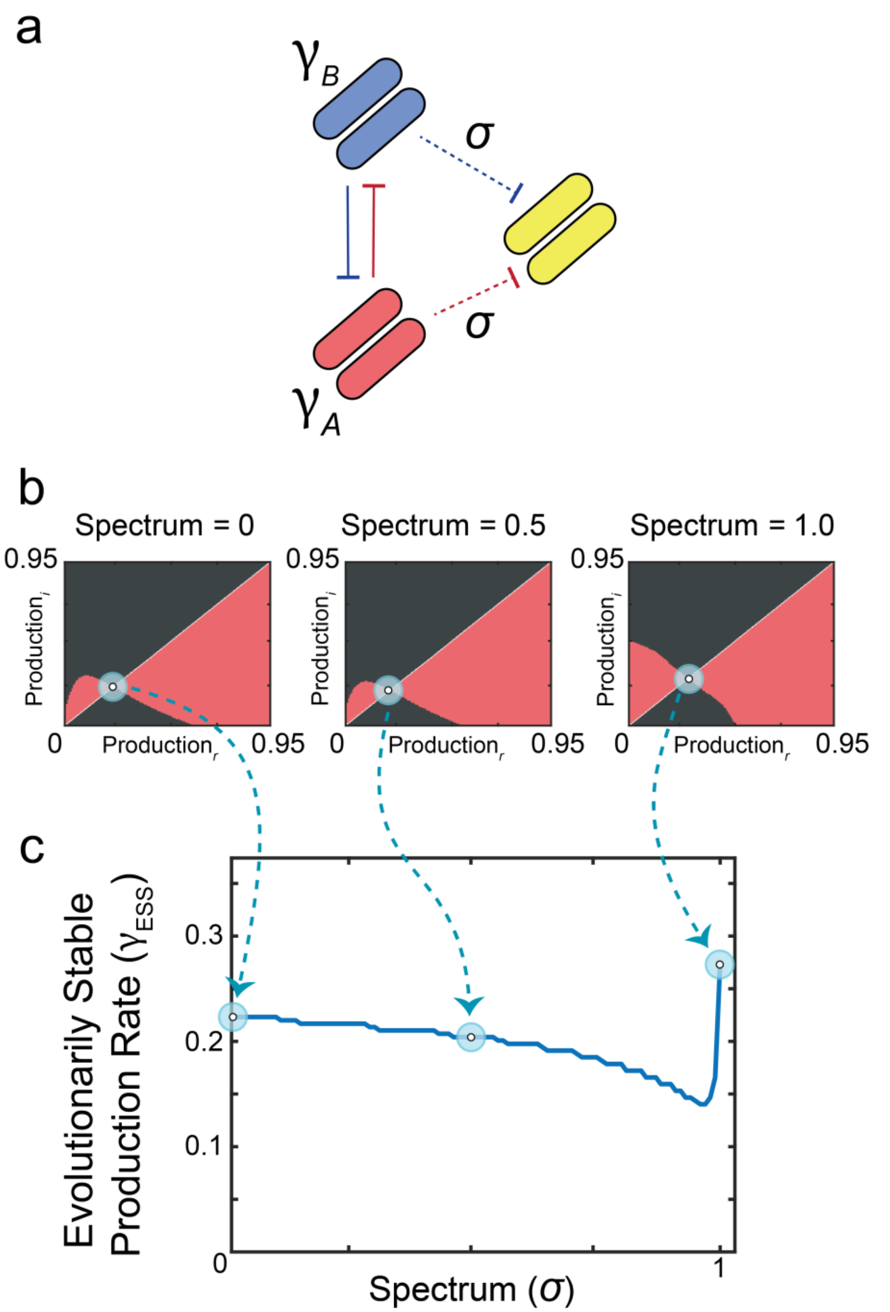

Figure S1 Identifying ESS toxin production rate across spectrum values. a) Ecological scenario, where both the focal strain (red) and the conspecific strain (blue) produce a toxin with equal spectrum. Each species is then able to explore all parameter space for production rate (γ = 0 to γ = 0.95) to find an evolutionarily stable production rate. b) Pairwise-invasion plots (PIPs) from competitions of the focal strain with production rate (γ_A) and conspecific with production rate (γ_B). Three example PIPs provided across the range of toxin spectra: σ = 0, σ = 0.5, σ = 1.0 All other parameters default, using density dependent degradation for toxin abundance (equations 8 and 9). c) Line-plot of ESS production rate (γ_{ESS}) as a function of spectrum (σ).

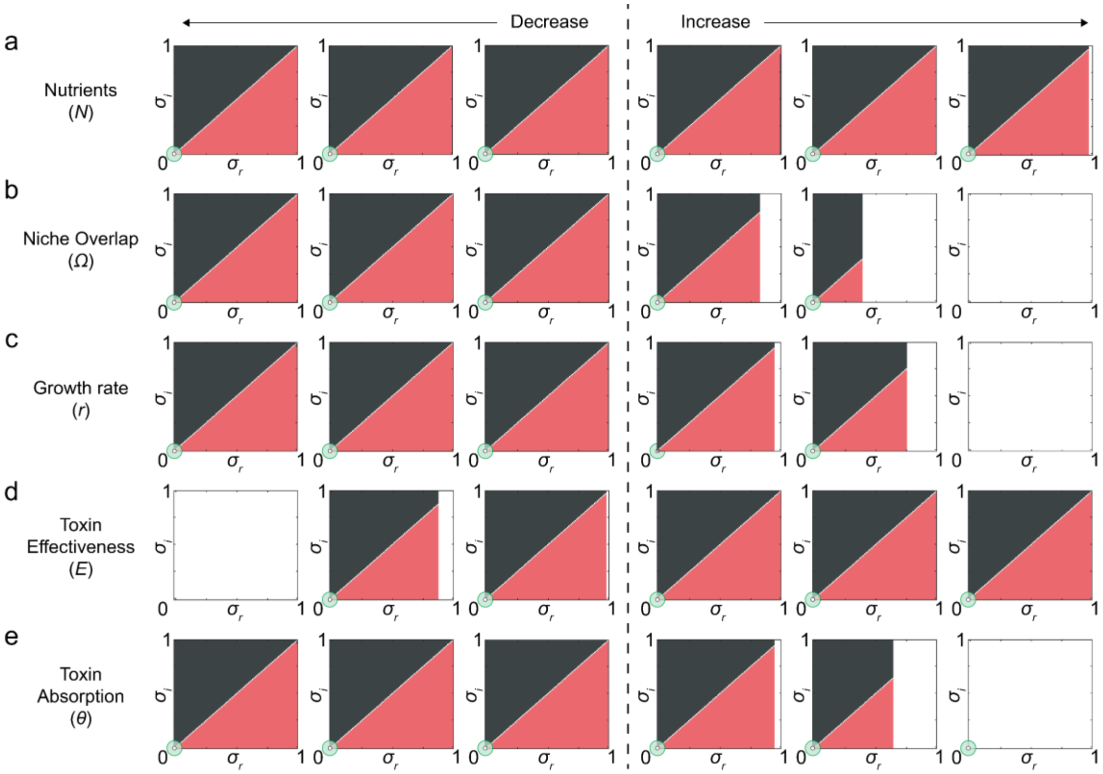

Figure S2 The prediction that narrow spectrum toxins evolve is robust to changes in parameters. Pairwise invasion plots are shown for spectrum of activity (σ) under density-dependent toxin degradation. Parameters are varied from default settings and spectrum evolutionary stable strategy (σ_{ESS}) is calculated (green circle) in each case. Across all conditions, the evolutionarily stable strategy is fully narrow (σ_{ESS} = 0). Parameter values are listed from left to right. The dashed vertical line indicates the position of default parameter values (**Table S3**). a) Starting nutrients. N_1 = N_2 = 0.2, 0.5, 0.75, 2.5, 5, 10;. b) Niche overlap. Ω_1 = 0.01, 0.05, 0.1, 0.5, 0.65, 0.75. c) Growth rate. r = 0.5, 0.7, 0.9, 1.5, 2, 2.5. d) Toxin effectiveness. E = 1, 7.5, 10, 20, 25, 30. e) Toxin absorption. θ = 0.1, 0.25, 0.5, 2, 3.5, 5. Sections of white in PIPs denote values for σ where the production rate ESS is zero (γ_{ESS} = 0), and therefore, there is no toxin production and no selection on σ. All parameters default unless otherwise indicated.

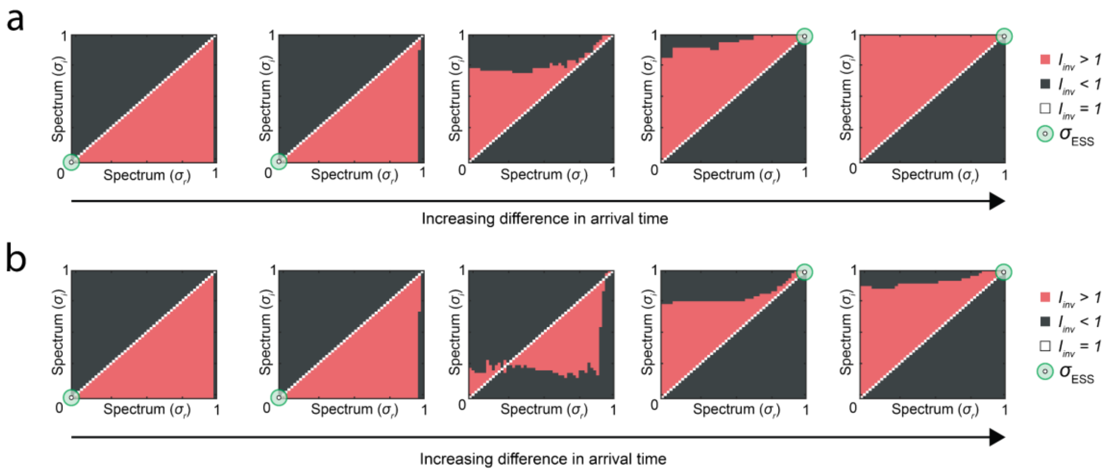

Figure S3 Pairwise invasibility plots (PIPs) for spectrum of activity (σ) with density-dependent toxin degradation and differences in arrival time for the two strains, using two different methodologies for modelling arrival time differences (Methods). In both, small differences in arrival time favours narrow-spectrum toxins, while large differences in arrival time favours broad-spectrum toxins. There is a transition zone for intermediate differences in arrival time where one sees more complex patterns and intermediate ESSs. This behaviour is only seen for a narrow range of parameters and we do not consider it further here. a) Differences in arrival time modelled by differences in initial abundance (also used in **Figure 4** and **Figure S5)**. The early arriving strain of the focal species always arrives at relative abundance of 1.0, and the community arrives halfway between the early and late arriving strains of the focal species. The initial relative abundance of the late arriving strain for each PIP is provided, from left to right: 0.9, 0.7, 0.5, 0.3, 0.1. b) Differences in arrival time explicitly modelled, where the early arriving strain is allowed to grow and produce toxins prior to the arrival of competitors. The initial relative abundance of all strains = 1.0. The community always arrives halfway between the early arriving strain and the late arriving strain of the focal species. The late arriving strain arrives once the early arriving strain reaches the following relative abundance, from left to right: 1.1, 1.3, 1.5, 1.7, 1.9. All parameters default unless otherwise indicated.

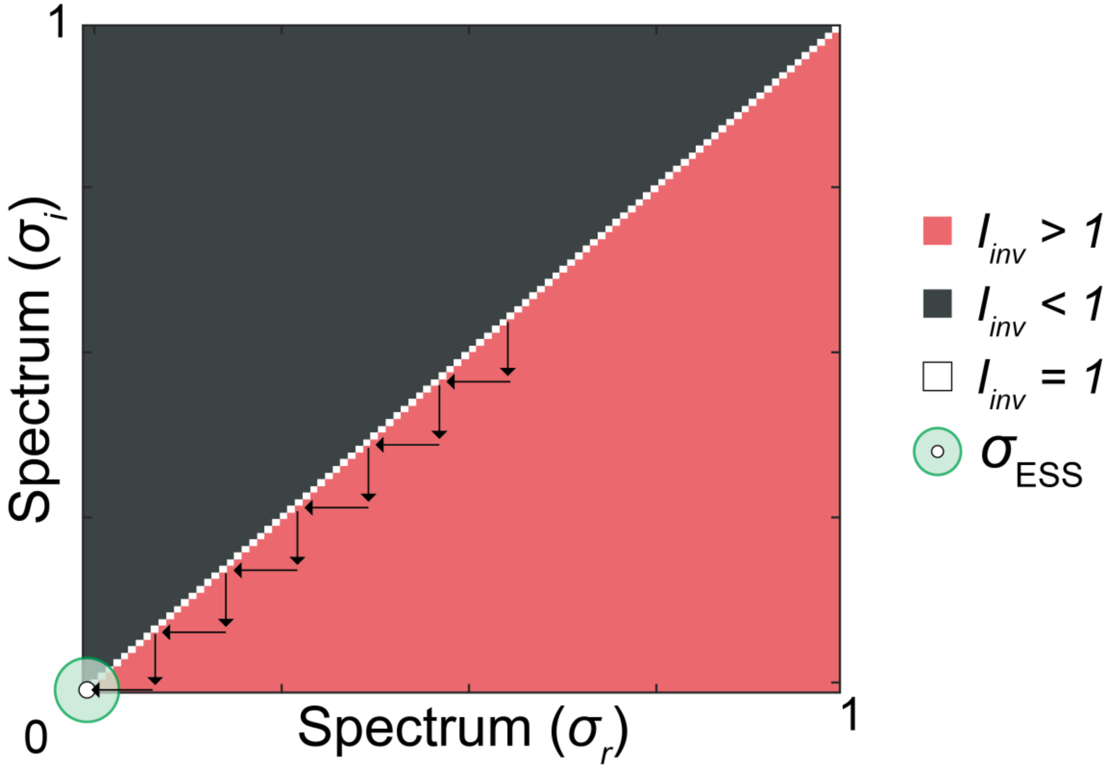

Figure S4 Pairwise invasibility plot for spectrum of activity (σ) with density-dependent toxin degradation and using equations 11 and 12 that incorporate a term for toxin loss driven by the producer. This change in assumption does not affect the key prediction that narrow-spectrum toxins will evolve. The main notable effect is that at the σ_{ESS} of 0, the investment in toxin production is slightly reduced i.e. γ_{ESS} is 0.21, vs γ_{ESS} = 0.22 in Figure 3. All parameters default unless otherwise indicated.

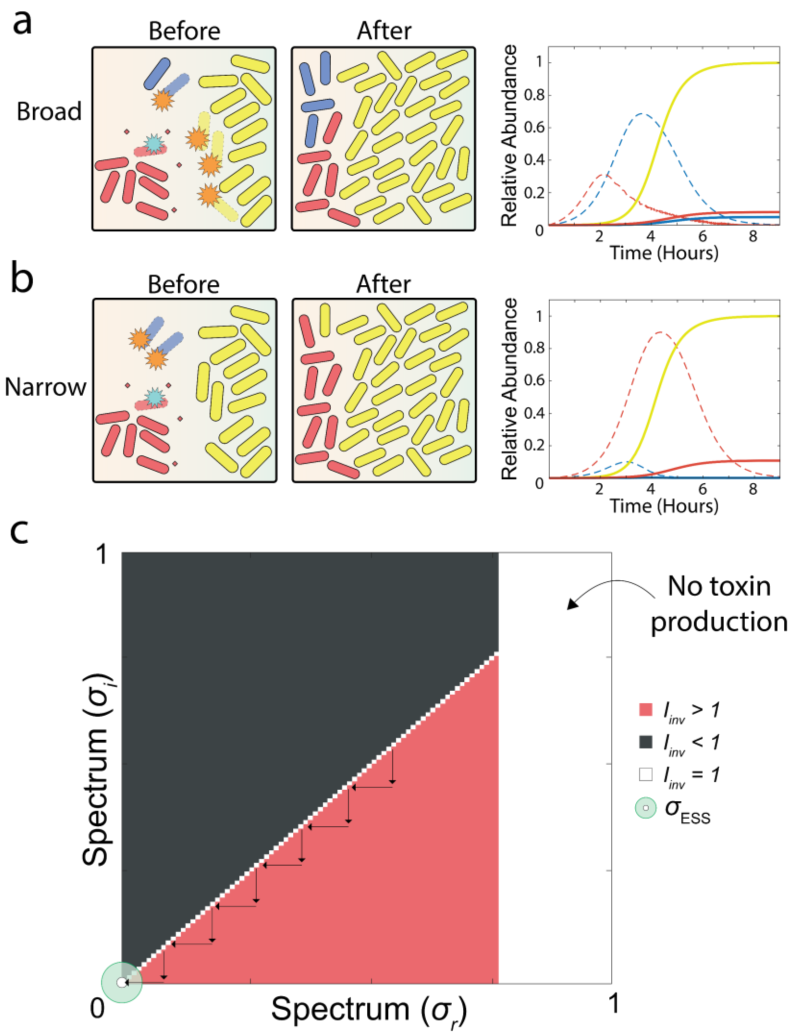

Figure S5 When the community greatly outnumbers the focal species, narrow spectrum toxins are favoured. In this model, like that shown in Figure 4, there is variability in arrival time of the focal strain. However, unlike that model, here we assume that there is always a large number of community cells present from the beginning. Cases where the focal strain arrives first drive its evolutionary dynamics, as this is when the great majority of cells are made. For this reason, we only show this case in the first two panels, but all cases are included in the calculation of the ESS. **a)** The focal strain (red) arrives before the conspecific (blue), produces broad-spectrum toxins, yet the community species (yellow) is too abundant to be overcome. The final pane provides representative temporal dynamics for initial relative abundances $A = 1.0$; $B = 0.4$; $C = 3.0$; and other parameters $\sigma_A = 1.0$; $\sigma_B = 0$; $\gamma_A = \gamma_B = 0.03$ (γ_{ESS} for $\sigma = 0$). **b)** Focal strain (red) arrives before the conspecific strain (blue) and produces a narrow-spectrum toxin focused on the conspecific strain, allowing the focal strain to outcompete the conspecific. Representative dynamics are shown for initial relative abundances of $A = 1.0$; $B = 0.4$; $C = 3.0$; and other parameters $\sigma_A = 0$; $\sigma_B = 0$; $\gamma_A = \gamma_B = 0.03$ (γ_{ESS} for $\sigma = 0$). For these illustrative plots, single species abundances are normalized to a maximum abundance of 1.0, and combined toxin abundances are normalized to a maximum of 1.0, which allows the different dynamics to be easily seen. **c)** Pairwise invasibility plot for spectrum of activity (σ) with density-dependent toxin degradation and variation in arrival time. The evolutionary stable spectrum strategy is fully narrow ($\sigma_{ESS} = 0$). For this plot, initial relative abundances are early arrival: 1.0; late arrival: 0.4; and community abundance: 3.0. All parameters default unless otherwise indicated.

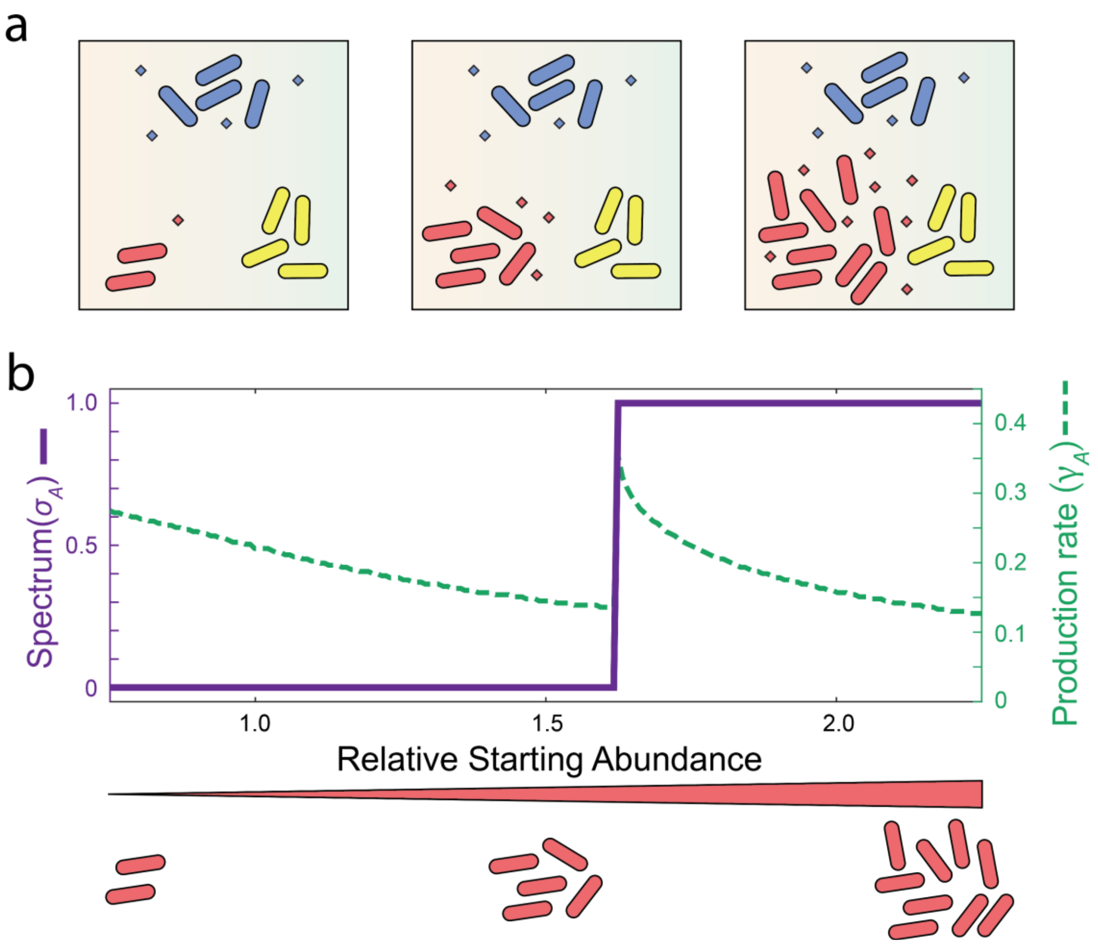

Figure S6 Modelling toxin evolution when the focal strain is the only member of its species in the patch. This scenario again predicts that broad-spectrum toxins are favoured at high abundance. a) Cartoons illustrating the initial conditions explored. **b**) Optimum strategy line plot. We identify the spectrum (σ_A, purple) and production rate (γ_A, green dotted) for the focal strain (red) that optimises its biomass, for a range of starting abundances. Niche competitor strategy: $\sigma_B = 0$; $\gamma_B = 0.22$. All other parameters default.

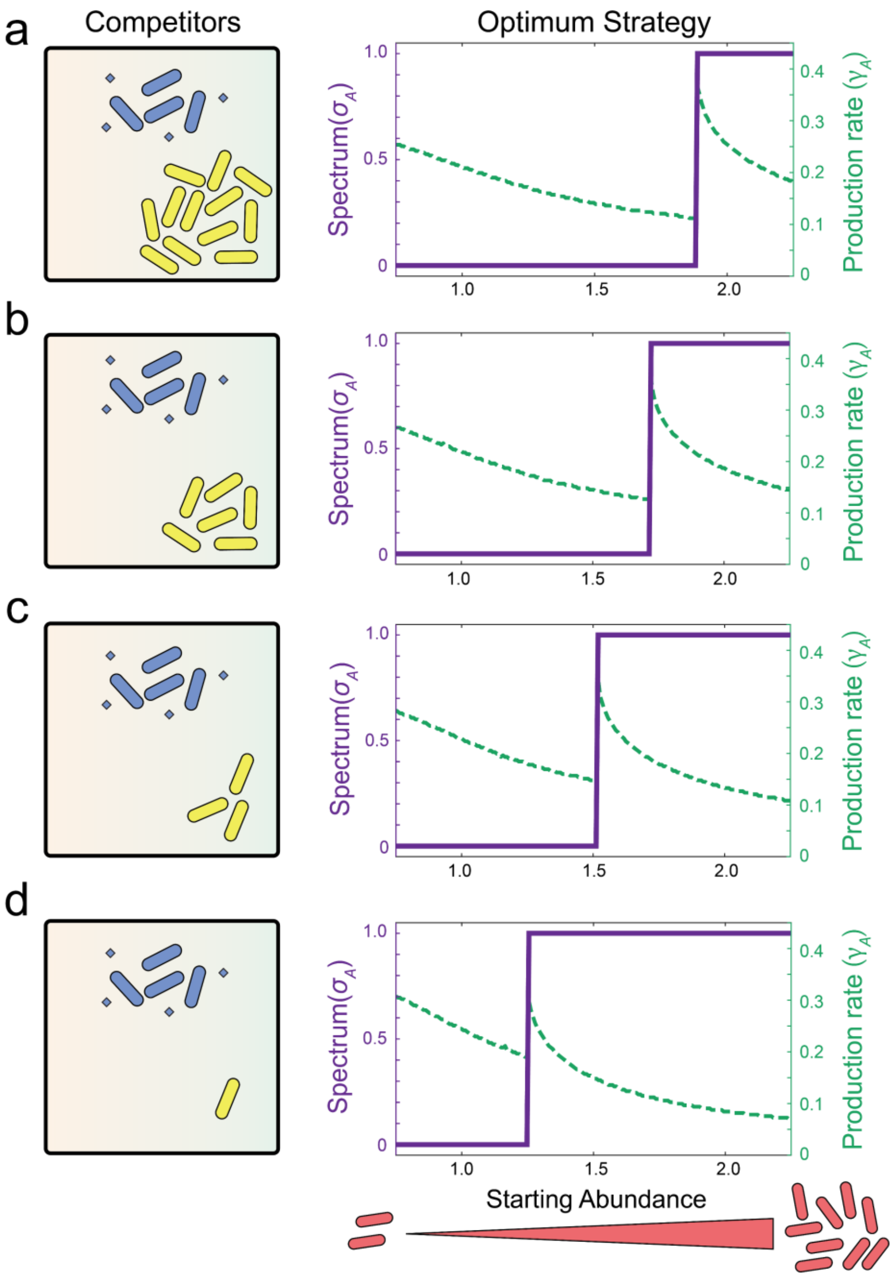

Figure S7 Modelling toxin evolution when the focal strain is the only member of its species in the patch (continued from **Figure S6**). Here we study the impacts of changing the starting abundance of the community (yellow). As community abundance goes up, the evolution of broad-spectrum toxins requires higher and higher abundances of the focal strain. Again, this supports the prediction that broad-spectrum toxins are most beneficial when locally abundant. Purple solid line denotes optimum spectrum, and the green dotted line denotes optimum production rate. **a)** Community starting relative abundance: 1.75. Niche competitor strategy: σ_B = 0; γ_B = 0.21. **b)** Community starting relative abundance: 1.25. Niche competitor strategy: σ_B = 0; γ_B = 0.22. **c)** Community starting relative abundance: 0.75. Niche competitor strategy: σ_B = 0; γ_B = 0.23. **d)** Community starting relative abundance: 0.25. Niche competitor strategy: σ_B = 0; γ_B = 0.24. Relative abundances normalised to starting abundance of the niche competitor species (*B*), which is 1.0 in all four plots. All parameters default unless otherwise indicated.

Table S1

Spectrum: 1 – intraclass; 2 – intraphylum; 3 – interphyla

Regulation: [N] - Nutrients. [S] - SOS response. [D] - Density-dependent. [O] – Other

Organism	Toxin	Regulator	Spectrum	Target	Notes
Escherichia coli	Colicin A	LexA [S] (*10*)	1 (*11*, *12*)	BtuB (*13*)	Originally isolated from *Citrobacter freundii* but primarily studied in *E. coli*.
	Colicin B	LexA [S] (*10*)	1 (*12*)	FepA (*13*)	
	Colicin D	LexA [S] (*10*)	1 (*12*)	FepA (*13*)	
	Colicin E1	LexA [S] (*10*)	1 (*12*)	BtuB (*13*)	
	Colicin E2	LexA [S]; AsnC [N] (*10*, *14*)	1 (*12*)	BtuB (*13*)	(*15*) – Provides discussion on specific O-antigen required for activity. Yet Ref. (*12*) shows activity outside of *E. coli*.<br><br>AsnC is a repressor, and is itself repressed under N-limiting conditions(*16*).
	Colicin E3	LexA [S] (*10*)	1 (*12*)	BtuB (*13*)	
	Colicin E5	LexA [S]; AsnC [N] (*14*)	1 (*12*)	BtuB (*13*)	
	Colicin E6	LexA [S]; AsnC [N] (*10*, *14*)	1 (*12*)	BtuB (*13*)	
	Colicin E7	LexA [S] (*10*)	1 (*12*)	BtuB (*13*)	
	Colicin E8	LexA [S]; AsnC [N] (*14*)	1 (*12*)	BtuB (*13*)	
	Colicin E9	SOS [S] (*17*)	1 (*12*)	BtuB (*13*)	
	Colicin Ia	LexA [S] (*10*)	1 (*12*)	Cir (*13*)	
	Colicin Ib	LexA [S] (*10*)	1 (*12*)	Cir (*13*)	
	Colicin K	LexA [S]; IscR [N] (*10*, *14*)	1 (*12*)	Tsx (*13*)	
	Colicin M	LexA [S] (*18*)	1 (*12*, *19*, *20*)	FhuA (*13*)	Some question here regarding amount of LexA regulation. A different strain was not able to be induced by MitC.
	Colicin N	LexA [S] (*10*)	1 (*12*)	OmpF; LPS (*13*, *21*)	

	Colicin U	LexA [S] (*10*)	1 (*15*)	OmpA (*13*)	Specific O-antigen required for activity.
	Colicin Y	LexA [S] (*10*)	1 (*15*)	OmpA (*22*)	
	Cloacin DF13	LexA [S] (*10*)	1 (*23*)	IutA (*13*)	Also isolated from and active against *Enterobacter cloacae*.
	Colicin 5	LexA [S] (*10*)	1 (*24*)	Tsx (*13*)	
	Colicin S4	LexA [S] (*10*)	1 (*12*)	OmpW (*25*)	
	Colicin R	LexA [S] (*15*)	1 (*15*)	OmpA (*15*)	O-antigen specific.
	Colicin Z	LexA [S] (*26*)	1 (*26*)	CjrC (*26*)	
	Microcin B17	OmpR [D] (*27*)	1 (*28*, *29*)	OmpF, SbmA (*30*)	Data with EnvZ and OmpR mutants showed that MccB17 regulation mirrors that of OmpC and is opposite to that of OmpF. Current model of OmpC/OmpF regulation proposes high osmolarity results in upregulation of OmpC.<br><br>Cessation of growth induced production (*31*). OmpR = stimulator (*32*). RpoS independent (referred to as KatF at time of publication(*32*).<br><br>Not considered density-dependent in the “Strict” analysis
	Microcin C	RpoS [D]; CRP [N]; H-NS [N]; Lrp [N] (*33*)	3 (*28*)	YejABEF (*34*) *“OmpF and other unidentified transport systems" (*35*)	*Was not able to corroborate OmpF as the Outer Membrane receptor from empirical data, though data in support of this is discussed in (*35*).
	Microcin J25	ppGpp [N], Lrp [N], IHF [N] (*36*)	1 (*37*)	FhuA (*37*)	Deletions of RpoS or CRP resulted in major decreases in MccJ25 production. The dynamic range between exponential and stationary phase expression was not affected, however, and so the authors conclude these two transcriptional regulators as not playing a role.
	Microcin V	Fur [N] (*38*)	1 (*39*)	Cir (*38*)	
	Microcin H47	Fur [N], MchX [O] (*40*, *41*)	1 (*42*)	FepA, Cir, Fiu, IroN (*40*, *43*)	MchX proposed to upregulate its own production and that of downstream genes.
	Microcin I47	Fur [N] (*44*)	1 (*44*)	FepA, Cir, Fiu, IroN (*40*, *45*)	This toxin has not been the direct focus of any studies to date, but it is quite clear from (*45*) that it will be the same as Microcin H47.

	Microcin M	Fur [N]<br>(*40*)	1<br>(*43*)	FepA, Cir, Fiu, IroN<br>(*40*, *43*)	
	Microcin N /<br>Microcin 24	Fur [N],<br>RpoS [D],<br>MdbA [O]<br>(*46*)	1<br>(*46*)	ManXYZ*<br>(*46*, *47*)	MdbA appears to be positive feedback transcription factor. *Early work refers to it as SemA. Later work says this is probably due to interference of ManXYZ by the Tn in SemA. Translocation across the outer membrane is TonB dependent(*40*).
	Microcin PDI	OmpR [D]<br>(*48*)	1<br>(*49*)	OmpF<br>(*50*)	Regulation of MccPDI production is clearly EnvZ/OmpR dependent, however, it appears to mirror the regulation of OmpF, where high osmolarity conditions result in low transcription levels. This is the opposite pattern of MccB17.<br><br>While the regulation appears density-dependent, it is being upregulated at low density, and therefore is not classified as “density-dependent” in the meta-analysis, which assumes upregulation at high density.
Shigella sonnei	Colicin J_s	LexA [S]<br>(*10*)	1<br>(*51*)	CjrC<br>(*52*)	
Klebsiella pneumoniae	Microcin E492	Fur [N];<br>MceX [O]<br>(*53*)	1<br>(*43*)	FepA, Cir, Fiu, IroN<br>(*43*)	
	Klebicin A1	SOS [S]<br>(*13*, *54*)	1<br>(*54*)	IutA<br>(*54*)	At time of receptor study, IutA had not been named. It is the ferric aerobactin receptor.
	Klebicin A2	SOS [S]<br>(*13*, *54*)	1<br>(*54*)	IutA<br>(*54*)	
	Klebicin A3	SOS [S]<br>(*13*, *54*)	1<br>(*54*)	IutA<br>(*54*)	
Yersinia pestis	Pesticin	LexA [S]<br>(*10*)	1<br>(*55*)	FyuA<br>(*56*)	FyuA: yersiniabactin receptor. FyuA expressed from *E. coli* implies Yersiniabactin producing strains would be susceptible. Non-yersiniabactin producing *E. coli* is resistant.
Yersinia frederiksenii	Colicin F_Y	LexA [S]<br>(*57*)	1<br>(*58*)	YiuR<br>(*58*)	
Yersinia enterocolitica	Enterocoliticin	SOS [S]<br>(*59*)	1<br>(*59*)	LPS<br>(*59*)	
Serratia marcescens	Bacteriocin 28B	SOS [S]<br>(*60*)	1<br>(*61*)	OmpF; OmpA<br>(*62*)	RegC regulated 28b expression, and RegC is LexA dependant.

Serratia plymuthica	Pyrrolnitrin	SplR [D] (LuxR homolog) (*63*)	3 (fungal) (*63*)	Diffusion (*64*, *65*)	Inhibitory activity against bacteria, fungus, human cell lines, all similar values. See also *Pseudomonas fluorescens*.
Pseudomonas aeruginosa	Pyocyanin	*las* [D], *rhl* [D], *pqs* [D] (*66*)	3 (*67*)	Diffusion	ROS-mediated damage. Free diffusion
	Pyocin R1	SOS [S] (*68*)	2 (*69*)	Lipopolysaccharide (*70*)	Activity against *Neisseria*.
	Pyocin R2	RecA [S]; PrtNR [S] (*71*)	2 (*72*)	Lipopolysaccharide (*70*)	Spectrum reference shows 1 out of 47 *Burkholderia* susceptible (*B. Cenocepacia*)
	Pyocin R5	SOS [S] (*68*)	3 (*68*, *72*)	Lipopolysaccharide (*70*)	Activity v *Campylobacter*. Absorption of Pyocin R5 by susceptible species while resistant did not absorb.. 4 out of 47 *Burkholderia* susceptible (3 - *B. Cenocepacia*; 1 *B. vietnamiensis*)
	Pyocin C*	SOS [S] (*73*, *74*)	2 (*73*, *74*)	LPS/LOS (*73*, *74*)	Activity against *Neisseria, Haemophilus, Pseudomonas*. Insufficient evidence to support Pyocin C being a single toxin. Instead, it appears Pyocin C might be a cocktail. Purification method from the 1970s from a Wild-Type culture.<br><br>*Not enough data to include in Figure 5 or the statistical analysis.
	Pyocin F1	SOS [S] (*75*)	1 (*76*, *77*)	LPS (*76*)	
	Pyocin F2	SOS [S] (*75*)	1 (*76*, *77*)	LPS (*76*)	
	Pyocin F3	SOS [S] (*77*)	1 (*76*, *77*)	LPS (*76*)	
	Pyocin S5*	Literature is conflicted	1 (*78*)	FptA (*79*)	Lacks regulation homology to known PrtNR/RecA. Multiple reports state that OxyR regulates the Pyocin S5 immunity protein based on experimental evidence, but no references actually lead to this experiment. One microarray study says oxidative stress leads to a putative S5 upregulation, along with other pyocin genes, and specifically does not rule out that the regulator could be DNA damage related(*80*). Apparently biofilm conditions and ceftazidime (B-lactam/cephalosporin)

					exposure also result in upregulation.<br><br>*Not enough evidence to include in Figure 5 or statistical analysis.
	Pseudomonas quinolone signal (PQS) / HQNO	GacA [D]<br>(*81*, *82*)	3<br>(*83*)	None*	Ligand-receptor interaction with MvfR / PqsR (transcription factor).<br>*Does not require receptor for activity
	PaeM	SOS [S]<br>(*84*)	1<br>(*85*)	FiuA ; TonB1<br>(*86*)	
	Hydrogen Cyanide	RhlR [D]<br>(*87*)	3<br>(*88*)	Free Diffusion<br>(*88*)	Regulator is quorum sensing homoserine lactone regulation.
Pseudomonas syringae	Syringacin M	SOS [S]<br>(*89*)	1<br>(*89*)	TonB-dependent ferrichrome transporter<br>(*89*, *90*)	
Pseudomonas fluorescens	Pyrrolnitrin	RpoS [D]<br>(*91*)<br><br>GacA [D]<br>(*92*)	3 (fungal)<br>(*91*)	Diffusion<br>(*64*, *65*)	RpoS is more closely linked with density-dependent regulation in *Pseudomonas* as compared to *E. coli*(*93*).<br><br>GacA is a global regulation implicated in biofilm formation but also many other processes(*81*).<br><br>Not considered density-dependent in the "Strict" analysis
Pseudomonas putida	LlpA$_{BW}$<br>(Putidacin L1)	RecA [S],<br>RecJ [S],<br>SpoT [N]<br>(*94*)	1<br>(*95*)	D-rhamnose containing O-antigen of LPS (or CPA)<br>(*96*)	
Burkholderia cenocepacia	BceTMilo	SOS [S]<br>(*72*)	2<br>(*72*)	LPS<br>(*72*)	
Burkholderia lata	Pyrrolnitrin	CepR [D]<br>(*97*)	3<br>(*98*)	Diffusion<br>(*64*, *65*)	CepR is a positive quorum sensing regulator (AHL-dependent) and seems also to control production of Pyrrolnitrin in some *Serratia* and *Pseudomonas*, though not experimentally demonstrated.
Chromobacterium violaceum	Violacein	LuxI [D]<br>/LuxR [D]<br>(*99*)	3 (fungal)<br>(*100*)	Cytoplasmic membrane<br>(*101*, *102*)	It remains an open question whether or not violacein should be considered a toxin, though it meets our requirements for inclusion and so it is included.
Lactobacillus plantarum	Plantaricin E/F<br>(Class IIb)	PlnC [D];<br>PlnD [D]	1<br>(*104*)	CorC<br>(*105*)	Three-component regulation, detecting PlnA, a peptide pheromone, which attaches

		(*103*)			to surface PlnB and activates PlnCD, resulting in rather canonical quorum-sensing.
	Plantaricin J/K (Class IIb)	PlnC [D]; PlnD [D] (*103*)	1 (*106*)	Putative APC transporter (*106*, *107*)	
Lactococcus lactis	Lacticin Q*	LnqR [O] (*108*)	3 (*109*)	No receptor required (*110*)	LnqR = TetR-like<br>LnqR is a positive regulator, is attenuated at higher temps, and therefore bacteriocin production decreases as temp increases.<br><br>*Excluded from Figure 5 and statistical analysis.
	Nisin	NisRK [D] (*111*)	3 (*112*)	Anionic lipid membrane ; Lipid II ; UDP (*113–115*)	There is an exogenous protein, NisP anchored to the exterior of the cell wall, necessary for NisRK mediated activation of Nisin production.<br><br>Nisin active against Gram negative bacteria if access to cytoplasmic membrane is obtained.
Bacillus subtilis	Subtilin	σH [D]; SpaR [D] (*116*, *117*)	3 (*118*)	Lipid II ; UDP (*115*, *119*)	Not considered density-dependent in the “Strict” analysis
Bacillus lichenformis	Bacitracin	AbrB [D,N]; Spo0A [D,S,N] (*120*)	3 (*121*, *122*)	C_{55}-isoprenyl pyrophosphate (*123*)	AbrB is a global regulator involved in the transition to stationary phase in *B. subtilis*.<br><br>Not considered density-dependent in the “Strict” analysis
Paenibacillus polymyxa	Polymyxin B	AbrB [D,N]; Spo0A [D,S,N] (*124*)	3 (*125*, *126*)	Anionic OM of Gram negative bacteria* (*126*, *127*)	Spo0A is produced starting in 2nd half of exponential phase and continues until start of stationary phase (*128*). It is also a master regulator for entering sporulation, activated by a myriad of signals including DNA damage, cell density, nutrient deprivation, Krebs cycle, and DNA synthesis (*129*).<br><br>*Some Gram positive bacteria absorb toxin and are sensitive.<br><br>1 amino acid different than Colistin (Polymyxin E).<br><br>Not considered density-dependent in the “Strict” analysis
	Colistin (Polymyxin E)	AbrB* [D,N];	3 (*125*, *126*)	Anionic OM of Gram negative bacteria**	*Implied from homology to Polymyxin B.

		Spo0A D,S,N (*124*)		(*126*, *127*)	**Some Gram positive bacteria absorb and are sensitive.<br><br>Not considered density-dependent in the "Strict" analysis
Staphylococcus epidermidis	Epidermin	Agr D;EpiP (*130*)	3 (*131*)	Lipid II (*132*)	Only 1 amino acid different than Gallidermin, with essentially identical minimum inhibitory concentrations.<br><br>Agr regulates EpiP, which is an extracellular protease that cleaves pre-epidermin into mature epidermin, an essential post-translational modification for activity.
Microbispora corallina	Microbisporicin	MibR D; MibW D; σ^{MibX} D; RelA N (*133*, *134*)	3 (*135*)	None* (*135*)	*Cell wall biosynthesis inhibition.<br>Active against nearly everything, Gram positive and Gram negative bacteria, though *Enterobacteriaceae* were resistant.<br><br>relA is ppGpp synthetase gene. σ^{MibX} is an Extracellular sigma factor. MibA is the structural bacteriocin gene. It (and 6 other genes in this operon) is regulated by MibR. 13 other Mib genes regulated by σ^{MibX}. (*134*) proposes a model which still requires high local density of producers.
Streptomyces coelicolor	Actinorhodin (ACT)	ActII-ORF4 D,S,N (*136*)	3 (*137*)	Numerous* (*137*)	ActII-ORF4 is regulated by at least 8 regulatory proteins (AdpA; LexA; AbsA2; DasR; DraR; AfsQ1; AtrA; ROK7B7). ActII-ORF4 translation dependent upon *bldA*. AdpA is growth dependent and triggers mycelium formation. *bldA* encodes tRNA for a rare codon and is only expressed in stationary phase.<br><br>*Mechanistic targets and receptor both unknown, however they claim that toxic activity is due to more than just oxidative damage<br><br>Not considered density-dependent in the "Strict" analysis
	Methylenomycin (MM)	MmyR D; MmfR D (*136*)	3 (*138*)	Peptidoglycan (*139*)	MMF autoregulator molecule = Quorum Sensing.

Streptomyces venezuelae	Chloramphenicol	JadR1 [D]; N/C starvation N (*140*–*142*)	3 (*143*, *144*)	Cytoplasmic access via diffusion (*145*, *146*)	JadR1 is behaving as a quorum system and strongly influences chloramphenicol production.
Streptomyces griseus	Streptomycin	AdpA [D] (*147*)	3 (*148*)	Diffusion; MscL (*149*, *150*)	AdpA responds to hormone A-factor in a density dependent manner.

Table S2

	**binaryPGLMM**			
	Coefficient	Std. error	*Z*-score	*p*-value
**Main**				
intercept	-2.0793	1.2823	-1.6215	0.1049
Spectrum - 2	-17.834	8404.8	-0.0021	0.9983
Spectrum - 3	4.1699	1.2196	3.4191	0.0006
**Strict**				
intercept	-2.5241	1.0696	-2.3598	0.0183
Spectrum - 2	-1.798	3.0364	-0.5921	0.5538
Spectrum - 3	2.9727	0.9944	2.9896	0.0028
	**MCMCglmm**			
	Posterior mean	95% Confidence Intervals	Effective Sample Size	pMCMC
**Main**				
intercept	-1.9417	-5.005 – 1.566	10973	0.2227
Spectrum - 2	-3.651	-11.19 – 2.975	10267	0.322
Spectrum - 3	5.523	2.792 – 8.456	10860	0.00007
**Strict**				
intercept	-3.3671	-7.555 – 0.809	9859	0.0812
Spectrum - 2	-3.5169	-11.05 – 3.383	10772	0.3567
Spectrum - 3	4.4655	1.3 – 8.255	1757	0.0015

Table S3 – Default Parameters

**Model parameter**	**Parameter Description**	**Default values**
$A, B, C\,(t = 0)$	Initial cell biomass of each strain	1e^{-4}
$N_1, N_2\,(t = 0)$	Initial pool of each nutrient	1.0
$T_A, T_B\,(t = 0)$	Initial biomass of each toxin	0
K_{N1}, K_{N2}	Saturation constant for nutrient uptake for each nutrient	5
r	Maximum growth rate	1
E	Killing efficiency of each toxin	15, 5*
L_T	Toxin degradation rate	0.75
h	Hill Coefficient	1
Ω_1	Niche Overlap (Species C consumption of Nutrient 1)	0.3
K_{min}	Minimum *K* value (strongest toxin-receptor affinity)	0.05
K_{max}	Maximum *K* value (weakest toxin-receptor affinity)	3
θ	Toxin Absorption	1

* Initial model (**Figure 2**)

SI References

1. J. D. Thompson, D. G. Higgins, T. J. Gibson, CLUSTAL W: Improving the sensitivity of progressive multiple sequence alignment through sequence weighting, position-specific gap penalties and weight matrix choice. *Nucleic Acids Res.* **22**, 4673–4680 (1994).
2. K. Tamura, M. Nei, Estimation of the number of nucleotide substitutions in the control region of mitochondrial DNA in humans and chimpanzees. *Mol. Biol. Evol.* **10**, 512–526 (1993).
3. S. Kumar, G. Stecher, M. Li, C. Knyaz, K. Tamura, MEGA X: Molecular evolutionary genetics analysis across computing platforms. *Mol. Biol. Evol.* **35**, 1547–1549 (2018).
4. A. R. Ives, T. Garland, in *Modern Phylogenetic Comparative Methods and their Application in Evolutionary Biology* (2014), pp. 231–261.
5. E. Paradis, J. Claude, K. Strimmer, APE: Analyses of Phylogenetics and Evolution in R language. *Bioinformatics*. **20**, 289–290 (2004).
6. J. D. Hadfield, MCMC Methods for Multi-Response Generalized Linear Mixed Models: The MCMCglmm R Package. *J. Stat. Softw.* **33**, 1–22 (2010).
7. A. Gelman, A. Jakulin, M. G. Pittau, Y. S. Su, A weakly informative default prior distribution for logistic and other regression models. *Ann. Appl. Stat.* **2**, 1360–1383 (2008).
8. L. McNally, M. Viana, S. P. Brown, Cooperative secretions facilitate host range expansion in bacteria. *Nat. Commun.* **5** (2014), doi:10.1038/NCOMMS5594.
9. H. C. Liedtke, H. Müller, J. Hafner, J. Penner, D. J. Gower, T. Mazuch, M. O. Rödel, S. P. Loader, Terrestrial reproduction as an adaptation to steep terrain in African toads. *Proc. R. Soc. B Biol. Sci.* **284** (2017), doi:10.1098/RSPB.2016.2598.
10. O. Gillor, J. A. C. Vriezen, M. A. Riley, The role of SOS boxes in enteric bacteriocin regulation. *Microbiology*. **154**, 1783–1792 (2008).
11. A. Marian, J. J. van Vught, J. de Graaff, A. H. Stouthamer, A genetic study of tolerance and resistance to colicin A in Citrobacter freundii. *Antonie Van Leeuwenhoek*. **41**, 309–318 (1975).
12. M. Feldgarden, M. A. Riley, High levels of colicin resistance in Escherichia coli. *Evolution (N. Y).* **52**, 1270–1276 (1998).
13. E. Cascales, S. K. Buchanan, D. Duche, C. Kleanthous, R. Lloubes, K. Postle, M. Riley, S. Slatin, D. Cavard, Colicin biology. *Microbiol. Mol. Biol. Rev.* **71**, 158–229 (2007).
14. S. Kamenšek, D. F. Browning, Z. Podlesek, S. J. W. Busby, D. Žgur-Bertok, M. Butala, Silencing of DNase Colicin E8 Gene Expression by a Complex Nucleoprotein Assembly Ensures Timely Colicin Induction. *PLOS Genet.* **11**, e1005354 (2015).
15. O. Rendueles, C. Beloin, P. Latour-Lambert, J. M. Ghigo, A new biofilm-associated colicin with increased efficiency against biofilm bacteria. *ISME J.* **8**, 1275–1288 (2014).
16. S. Poggio, C. Domeinzain, A. Osorio, L. Camarena, The nitrogen assimilation control (Nac) protein represses asnC and asnA transcription in Escherichia coli. *FEMS Microbiol. Lett.* **206**, 151–156 (2002).
17. S. Bano, M. Vankemmelbeke, C. N. Penfold, R. James, Pattern of induction of colicin E9 synthesis by sub MIC of Norfloxacin antibiotic. *Microbiol. Res.* **168**, 661–666 (2013).
18. K. Schaller, R. Dreher, V. Braun, "Structural and Functional Properties of Colicin M" (1981), (available at http://jb.asm.org/).
19. A. C. Graham, B. A. D. Stocker, Genetics of sensitivity of Salmonella species to colicin M and bacteriophages T5, T1, and ES18. *J. Bacteriol.* **130**, 1214–1223 (1977).
20. E. Łojewska, T. Sakowicz, A. Kowalczyk, M. Konieczka, J. Grzegorczyk, P. Sitarek, E. Skała, P. Czarny, T. Śliwiński, T. Kowalczyk, Production of recombinant colicin M in Nicotiana tabacum plants and its antimicrobial activity. *Plant Biotechnol. Rep.* **14**, 33–43 (2020).
21. Y. C. Kim, A. W. Tarr, C. N. Penfold, Colicin import into E. coli cells: A model system for insights into the import mechanisms of bacteriocins. *Biochim. Biophys. Acta - Mol. Cell Res.* **1843**, 1717–1731 (2014).
22. J. Bosák, L. Micenková, M. Doležalová, D. Šmajs, Colicins U and Y inhibit growth of Escherichia coli strains via recognition of conserved OmpA extracellular loop 1. *Int. J.*

Med. Microbiol. **306**, 486–494 (2016).
23. R. James, Molecular cloning and purification of Klebicin B. *J. Gen. Microbiol.* **134**, 2525–2533 (1988).
24. H. Yang, L. Wan, X. Li, H. Cai, L. Chen, S. Li, Y. Li, J. Cheng, X. Lu, High level expression of His-tagged colicin 5 in E. coli and characterization of its narrow-spectrum bactericidal activity and pore-forming action. *Protein Expr. Purif.* **54**, 309–317 (2007).
25. H. Pilsl, D. Smajs, V. Braun, Characterization of Colicin S4 and Its Receptor, OmpW, a Minor Protein of the Escherichia coli Outer Membrane. *J. Bacteriol.* **181**, 3578 (1999).
26. L. Micenková, J. Bosák, J. Kucera, M. Hrala, T. Dolejšová, O. Šedo, D. Linke, R. Fišer, D. Šmajs, Colicin Z, a structurally and functionally novel colicin type that selectively kills enteroinvasive Escherichia coli and Shigella strains. *Sci. Rep.* **9**, 1–12 (2019).
27. C. Hernandez-Chico, J. L. San Millan, R. Kolter, F. Moreno, Growth phase and OmpR regulation of transcription of microcin B17 genes. *J. Bacteriol.* **167**, 1058–1065 (1986).
28. I. A. Khmel, V. M. Bondarenko, I. M. Manokhina, E. I. Basyuk, A. Z. Metlitskaya, V. A. Lipasova, Y. M. Romanova, Isolation and characterization of *Escherichia coli* strains producing microcins of B and C types. *FEMS Microbiol. Lett.* **111**, 269–274 (1993).
29. C. Asensio, J. C. Pérez-Díaz, M. C. Martínez, F. Baquero, A new family of low molecular weight antibiotics from enterobacteria. *Biochem. Biophys. Res. Commun.* **69**, 7–14 (1976).
30. M. Lavina, A. P. Pugsley, F. Moreno, Identification, mapping, cloning and characterization of a gene (sbm A) required for microcin B17 action on Escherichia coli K12. *J. Gen. Microbiol.* **132**, 1685–1693 (1986).
31. N. Connell, Z. Han, F. Moreno, R. Kolter, An E. coli promoter induced by the cessation of growth. *Mol. Microbiol.* **1**, 195–201 (1987).
32. D. E. Bohannon, N. Connell, J. Keener, A. Tormo, M. Espinosa-Urgel, M. M. Zambrano, R. Kolter, Stationary-phase-inducible "'gearbox'" promoters: Differential effects of katF mutations and role of σ70. *J. Bacteriol.* **173**, 4482–4492 (1991).
33. D. Fomenko, A. Veselovskii, I. Khmel, Regulation of microcin C51 operon expression: The role of global regulators of transcription. *Res. Microbiol.* **152**, 469–479 (2001).
34. M. Novikova, A. Metlitskaya, K. Datsenko, T. Kazakov, A. Kazakov, B. Wanner, K. Severinov, The Escherichia coli Yej transporter is required for the uptake of translation inhibitor microcin C. *J. Bacteriol.* **189**, 8361–8365 (2007).
35. G. H. M. Vondenhoff, B. Blanchaert, S. Geboers, T. Kazakov, K. A. Datsenko, B. L. Wanner, J. Rozenski, K. Severinov, A. Van Aerschot, Characterization of peptide chain length and constituency requirements for YejABEF-mediated uptake of microcin C analogues. *J. Bacteriol.* **193**, 3618–3623 (2011).
36. M. J. Chiuchiolo, M. A. Delgado, R. N. Farías, R. A. Salomón, Growth-phase-dependent expression of the cyclopeptide antibiotic microcin J25. *J. Bacteriol.* **183**, 1755–1764 (2001).
37. D. Destoumieux-Garzón, S. Duquesne, J. Peduzzi, C. Goulard, M. Desmadril, L. Letellier, S. Rebuffat, P. Boulanger, The iron-siderophore transporter FhuA is the receptor for the antimicrobial peptide microcin J25: Role of the microcin Val11-Pro16 β-hairpin region in the recognition mechanism. *Biochem. J.* **389**, 869–876 (2005).
38. H. Chehade, V. Braun, "Iron-regulated synthesis and uptake of colicin V" (1988), , doi:10.1111/j.1574-6968.1988.tb02591.x.
39. L. J. Cohen, S. Han, Y. H. Huang, S. F. Brady, Identification of the Colicin V Bacteriocin Gene Cluster by Functional Screening of a Human Microbiome Metagenomic Library. *ACS Infect. Dis.* **4**, 27 (2018).
40. S. I. Patzer, M. R. Baquero, D. Bravo, F. Moreno, K. Hantke, The colicin G, H and X determinants encode microcins M and H47, which might utilize the catecholate siderophore receptors FepA, Cir, Fiu and IroN. *Microbiology*. **149**, 2557–2570 (2003).
41. E. Rodriguez, M. Lavina, Genetic analysis of microcin H47 immunity. *Can. J. Microbiol.* **44**, 692–697 (1998).
42. J. D. Palmer, B. M. Mortzfeld, E. Piattelli, M. W. Silby, B. A. McCormick, V. Bucci, Microcin H47: A Class IIb Microcin with Potent Activity against Multidrug Resistant Enterobacteriaceae. *ACS Infect. Dis.* **6**, 672–679 (2020).
43. G. Vassiliadis, D. Destoumieux-Garzon, C. Lombard, S. Rebuffat, J. Peduzzi, Isolation

and characterization of two members of the siderophore-microcin family, microcins M and H47. *Antimicrob. Agents Chemother.* **54**, 288–297 (2010).
44. M. E. Poey, M. F. Azpiroz, M. Lavina, Comparative analysis of chromosome-encoded microcins. *Antimicrob. Agents Chemother.* **50**, 1411–1418 (2006).
45. E. M. Nolan, C. T. Walsh, Investigations of the MceIJ-catalyzed posttranslational modification of the microcin E492 C-terminus: linkage of ribosomal and nonribosomal peptides to form "trojan horse" antibiotics. *Biochemistry*. **47**, 9289–9299 (2008).
46. G. J. O'Brien, "Molecular analysis of microcin 24 : Genetics, secretion and mode of action of a novel microcin" (1996), (available at https://core.ac.uk/download/pdf/35467757.pdf).
47. S. Bieler, F. Silva, C. Soto, D. Belin, Bactericidal activity of both secreted and nonsecreted microcin E492 requires the mannose permease. *J. Bacteriol.* **188**, 7049–7061 (2006).
48. Z. Zhao, L. H. Orfe, J. Liu, S. Y. Lu, T. E. Besser, D. R. Call, Microcin PDI regulation and proteolytic cleavage are unique among known microcins. *Sci. Rep.* **7** (2017), doi:10.1038/srep42529.
49. S. Y. Lu, T. Graça, J. J. Avillan, Z. Zhao, D. R. Call, Microcin PDI inhibits antibioticresistant strains of Escherichia coli and Shigella through a mechanism of membrane disruption and protection by homotrimer self-immunity. *Appl. Environ. Microbiol.* **85** (2019), doi:10.1128/AEM.00371-19.
50. Z. Zhao, L. J. Eberhart, L. H. Orfe, S. Y. Lu, T. E. Besser, D. R. Call, Genome-wide screening identifies six genes that are associated with susceptibility to Escherichia coli microcin PDI. *Appl. Environ. Microbiol.* **81**, 6953–6963 (2015).
51. J. Šmarda, J. Petrželová, B. Vyskot, Colicin Js of Shigella sonnei: Classification of type colicin "7." *Zentralblatt fur Bakteriol. Mikrobiol. und Hyg. - Abt. 1 Orig. A*. **263**, 530–540 (1987).
52. D. Šmajs, G. M. Weinstock, The iron- and temperature-regulated cjrBC genes of Shigella and enteroinvasive Escherichia coli strains code for colicin Js uptake. *J. Bacteriol.* **183**, 3958–3966 (2001).
53. A. E. Marcoleta, S. Gutiérrez-Cortez, F. Hurtado, Y. Argandoña, G. Corsini, O. Monasterio, R. Lagos, The Ferric uptake regulator (Fur) and iron availability control the production and maturation of the antibacterial peptide microcin E492. *PLoS One*. **13**, e0200835 (2018).
54. P. C. Cooper, R. James, Three immunity types of klebicins which use the cloacin DF13 receptor of Klebsiella pneumoniae. *J. Gen. Microbiol.* **131**, 2313–2318 (1985).
55. M. Elgat, R. Ben-Gurion, Mode of action of pesticin. *J. Bacteriol.* **98**, 359–367 (1969).
56. A. Rakin, E. Saken, D. Harmsen, J. Heesemann, The pesticin receptor of Yersinia enterocolitica: a novel virulence factor with dual function. *Mol. Microbiol.* **13**, 253–263 (1994).
57. J. Bosák, P. Laiblová, J. Šmarda, D. Dě, D. Šmajs, Novel Colicin F Y of Yersinia frederiksenii Inhibits Pathogenic Yersinia Strains via YiuR-Mediated Reception, TonB Import, and Cell Membrane Pore Formation (2012), doi:10.1128/JB.05885-11.
58. J. Bosák, L. Micenková, M. Vrba, A. Ševčíková, D. Dědičová, D. Garzetti, D. Šmajs, Unique Activity Spectrum of Colicin FY: All 110 Characterized Yersinia enterocolitica Isolates Were Colicin FY Susceptible. *PLoS One*. **8**, e81829 (2013).
59. E. Strauch, H. Kaspar, C. Schaudinn, P. Dersch, K. Madela, C. Gewinner, S. Hertwig, J. Wecke, B. Appel, Characterization of Enterocoliticin, a Phage Tail-Like Bacteriocin, and Its Effect on Pathogenic Yersinia enterocolitica Strains. *Appl. Environ. Microbiol.* **67**, 5634–5642 (2001).
60. S. Ferrer, M. B. Viejo, J. F. Guasch, J. Enfedaque, M. Regué, Genetic evidence for an activator required for induction of colicin-like bacteriocin 28b production in Serratia marcescens by DNA-damaging agents. *J. Bacteriol.* **178**, 951–960 (1996).
61. J. Enfedaque, S. Ferrer, J. F. Guasch, J. Tomás, M. Regué, Bacteriocin 28b from Serratia marcescens N28b: Identification of Escherichia coli surface components involved in bacteriocin binding and translocation. *Can. J. Microbiol.* **42**, 19–26 (1996).
62. J. F. Guasch, S. Ferrer, J. Enfedaque, M. B. Viejo, M. Regue, A17 kDa outer-membrane protein (Omp4) from Seruatia marcescens confers partial resistance to bacteriocin 28b when expressed in Escherichia coli. *Microbiology*. **141**, 2535–2542 (1995).

63. X. Liu, M. Bimerew, Y. Ma, H. Müller, M. Ovadis, L. Eberl, G. Berg, L. Chernin, Quorum-sensing signaling is required for production of the antibiotic pyrrolnitrin in a rhizospheric biocontrol strain of Serratia plymuthica. *FEMS Microbiol. Lett.* **270**, 299–305 (2007).
64. R. K. Tripathi, D. Gottlieb, Mechanism of Action of the Antifungal Antibiotic Pyrrolnitrin. *J. Bacteriol.* **100**, 310 (1969).
65. J. Kilani, S. Fillinger, Phenylpyrroles: 30 Years, Two Molecules and (Nearly) No Resistance. *Front. Microbiol.* **7**, 2014 (2016).
66. S. Higgins, S. Heeb, G. Rampioni, M. P. Fletcher, P. Williams, M. Cámara, Differential regulation of the phenazine biosynthetic operons by quorum sensing in Pseudomonas aeruginosa PAO1-N. *Front. Cell. Infect. Microbiol.* **8**, 252 (2018).
67. S. S. Baront, J. J. Rowe, "Antibiotic Action of Pyocyanin" (1981).
68. C. C. Blackwell, F. P. Winstanley, W. A. Telfer Brunton, Sensitivity of thermophilic campylobacters to R-type pyocines of Pseudomonas aeruginosa. *J. Med. Microbiol.* **15**, 247–251 (1982).
69. C. C. Blackwell, J. A. Law, Typing of non-serogroupable Neisseria meningitidis by means of sensitivity to R-type pyocines of Pseudomonas aeruginosa. *J. Infect.* **3**, 370–378 (1981).
70. T. Köhler, V. Donner, C. Van Delden, Lipopolysaccharide as shield and receptor for R-pyocin-mediated killing in Pseudomonas aeruginosa. *J. Bacteriol.* **192**, 1921–1928 (2010).
71. H. Matsui, Y. Sano, H. Ishihara, T. Shinomiya, Regulation of pyocin genes in Pseudomonas aeruginosa by positive (prtN) and negative (prtR) regulatory genes. *J. Bacteriol.* **175**, 1257–1263 (1993).
72. G. W. Yao, I. Duarte, T. T. Le, L. Carmody, J. J. LiPuma, R. Young, C. F. Gonzalez, A broadhost- range tailocin from Burkholderia cenocepacia. *Appl. Environ. Microbiol.* **83** (2017), doi:10.1128/AEM.03414-16.
73. F. K. N. Lee, K. C. Dudas, J. A. Hanson, M. B. Nelson, P. T. LoVerde, M. A. Apicella, The R-type pyocin of Pseudomonas aeruginosa C is a bacteriophage tail- like particle that contains single-stranded DNA. *Infect. Immun.* **67**, 717–725 (1999).
74. M. J. Filiatrault, J. Munson, A. A. Campagnari, Genetic analysis of a pyocin-resistant lipooligosaccharide (LOS) mutant of Haemophilus ducreyi: Restoration of full-length LOS restores pyocin sensitivity. *J. Bacteriol.* **183**, 5756–5761 (2001).
75. K. Kuroda, M. Kageyama, Biochemical properties of a new flexuous bacteriocin, pyocin Fl, produced by Pseudomonas aeruginosa. *J. Biochem.* **85**, 7–19 (1979).
76. S. Saha, thesis, University of Toronto (2016).
77. K. Kuroda, M. Kageyama, Comparative study on F-type pyocins of Pseudomonas aeruginosa. *J. Biochem.* **89**, 1721–1736 (1981).
78. H. Ling, N. Saeidi, B. H. Rasouliha, M. W. Chang, A predicted S-type pyocin shows a bactericidal activity against clinical Pseudomonas aeruginosa isolates through membrane damage. *FEBS Lett.* **584**, 3354–3358 (2010).
79. A. Elfarash, J. Dingemans, L. Ye, A. A. Hassan, M. Craggs, C. Reimmann, M. S. Thomas, P. Cornelis, Pore-forming pyocin S5 utilizes the FptA ferripyochelin receptor to kill Pseudomonas aeruginosa. *Microbiol. (United Kingdom)*. **160**, 261–269 (2014).
80. W. Chang, D. A. Small, F. Toghrol, W. E. Bentley, Microarray analysis of Pseudomonas aeruginosa reveals induction of pyocin genes in response to hydrogen peroxide. *BMC Genomics*. **6**, 1–14 (2005).
81. H. Huang, X. Shao, Y. Xie, T. Wang, Y. Zhang, X. Wang, X. Deng, An integrated genomic regulatory network of virulence-related transcriptional factors in Pseudomonas aeruginosa. *Nat. Commun.* **10**, 1–13 (2019).
82. S. Heeb, M. P. Fletcher, S. R. Chhabra, S. P. Diggle, P. Williams, M. Cámara, Quinolones: From antibiotics to autoinducers. *FEMS Microbiol. Rev.* **35** (2011), pp. 247–274.
83. L. M. Mashburn, M. Whiteley, Membrane vesicles traffic signals and facilitate group activities in a prokaryote. *Nature*. **437**, 422–425 (2005).
84. H. Barreteau, M. Tiouajni, M. Graille, N. Josseaume, A. Bouhss, D. Patin, D. Blanot, M. Fourgeaud, J. L. Mainardi, M. Arthur, H. Van Tilbeurgh, D. Mengin-Lecreulx, T. Touzé, Functional and structural characterization of PaeM, a colicin M-like bacteriocin produced

by Pseudomonas aeruginosa. *J. Biol. Chem.* **287**, 37395–37405 (2012).
85. H. Barreteau, A. Bouhss, M. Fourgeaud, J. L. Mainardi, T. Touzé, F. Gérard, D. Blanot, M. Arthur, D. Mengin-Lecreulx, Human- and plant-pathogenic Pseudomonas species produce bacteriocins exhibiting colicin M-like hydrolase activity towards peptidoglycan precursors. *J. Bacteriol.* **191**, 3657–3664 (2009).
86. L. Latino, D. Patin, D. Chérier, T. Touzé, C. Pourcel, H. Barreteau, D. Mengin-Lecreulx, Impact of FiuA outer membrane receptor polymorphism on the resistance of Pseudomonas aeruginosa toward Peptidoglycan Lipid II-Targeting PaeM pyocins. *J. Bacteriol.* **201** (2019), doi:10.1128/JB.00164-19.
87. M. K. Winson, M. CAMARAt, A. Latifi, M. Foglino, S. RAM CHHABRAt, Ma. DAYKINt, M. BALLYt, V. CHAPONt, G. P. C Salmond, B. W. BYCROFTt, A. LAZDUNSKIt, G. S. A B Stewart, P. WILLIAMSt, "Multiple N-acyl-L-homoserine lactone signal molecules regulate production of virulence determinants and secondary metabolites in Pseudomonas aeruginosa (autoinducers/quorum sensing/gene regulation)" (1995).
88. L. A. Gallagher, C. Manoil, Pseudomonas aeruginosa PAO1 kills Caenorhabditis elegans by cyanide poisoning. *J. Bacteriol.* **183**, 6207–6214 (2001).
89. R. Grinter, A. W. Roszak, R. J. Cogdell, J. J. Milner, D. Walker, The crystal structure of the lipid II-degrading bacteriocin syringacin M suggests unexpected evolutionary relationships between colicin M-like bacteriocins. *J. Biol. Chem.* **287**, 38876–38888 (2012).
90. M. G. K. Ghequire, L. Kemland, E. Anoz-Carbonell, S. K. Buchanan, R. De Mot, A natural chimeric Pseudomonas bacteriocin with novel pore-forming activity parasitizes the ferrichrome transporter. *MBio*. **8** (2017), doi:10.1128/mBio.01961-16.
91. A. Sarniguet, J. Kraus, M. D. Henkels, A. M. Muehlchen, J. E. Loper, The sigma factor σs affects antibiotic production and biological control activity of Pseudomonas fluorescens Pf-5. *Proc. Natl. Acad. Sci. U. S. A.* **92**, 12255–12259 (1995).
92. C. A. Whistler, N. A. Corbell, A. Sarniguet, W. Ream, J. E. Loper, The two-component regulators GacS and GacA influence accumulation of the stationary-phase sigma factor σ(s) and the stress response in Pseudomonas fluorescens Pf-5. *J. Bacteriol.* **180**, 6635–6641 (1998).
93. V. Venturi, Control of rpoS transcription in Escherichia coli and Pseudomonas: why so different? *Mol. Microbiol.* **49**, 1–9 (2003).
94. P. E. Los Santos, A. H. A. Parret, R. Mot, Stress-related *Pseudomonas* genes involved in production of bacteriocin LlpA. *FEMS Microbiol. Lett.* **244**, 243–250 (2005).
95. M. G. K. Ghequire, W. Li, P. Proost, R. Loris, R. De Mot, Plant lectin-like antibacterial proteins from phytopathogens Pseudomonas syringae and Xanthomonas citri. *Environ. Microbiol. Rep.* **4**, 373–380 (2012).
96. L. C. McCaughey, R. Grinter, I. Josts, A. W. Roszak, K. I. Waløen, R. J. Cogdell, J. Milner, T. Evans, S. Kelly, N. P. Tucker, O. Byron, B. Smith, D. Walker, Lectin-Like Bacteriocins from Pseudomonas spp. Utilise D-Rhamnose Containing Lipopolysaccharide as a Cellular Receptor. *PLoS Pathog.* **10**, e1003898 (2014).
97. S. Schmidt, J. F. Blom, J. Pernthaler, G. Berg, A. Baldwin, E. Mahenthiralingam, L. Eberl, Production of the antifungal compound pyrrolnitrin is quorum sensing-regulated in members of the Burkholderia cepacia complex. *Environ. Microbiol.* **11**, 1422–1437 (2009).
98. N. El-Banna, G. Winkelmann, Pyrrolnitrin from Burkholderia cepacia: Antibiotic activity against fungi and novel activities against streptomycetes. *J. Appl. Microbiol.* **85**, 69–78 (1998).
99. K. H. McClean, M. K. Winson, L. Fish, A. Taylor, S. R. Chhabra, M. Camara, M. Daykin, J. H. Lamb, S. Swift, B. W. Bycroft, G. S. A. B. Stewart, P. Williams, Quorum sensing and Chromobacterium violaceum: Exploitation of violacein production and inhibition for the detection of N-acylhomoserine lactones. *Microbiology*. **143**, 3703–3711 (1997).
100. H. C. Lichstein, V. F. Van De Sand, Violacein, an Antibiotic Pigment Produced by Chromobacterium Violaceum. *J. Infect. Dis.* **76**, 47–51 (1945).
101. A. C. G. Cauz, G. P. B. Carretero, G. K. V. Saraiva, P. Park, L. Mortara, I. M. Cuccovia, M. Brocchi, F. J. Gueiros-Filho, Violacein targets the cytoplasmic membrane of bacteria. *ACS Infect. Dis.* **5**, 539–549 (2019).
102. S. Y. Choi, S. Lim, G. Cho, J. Kwon, W. Mun, H. Im, R. J. Mitchell, Chromobacterium

violaceum delivers violacein, a hydrophobic antibiotic, to other microbes in membrane vesicles. *Environ. Microbiol.* **22**, 705–713 (2020).
103. D. B. Diep, R. Myhre, O. Johnsborg, Å. Aakra, I. F. Nes, Inducible bacteriocin production in Lactobacillus is regulated by differential expression of the pln operons and by two antagonizing response regulators, the activity of which is enhanced upon phosphorylation. *Mol. Microbiol.* **47**, 483–494 (2003).
104. E. L. Anderssen, D. B. Diep, I. F. Nes, V. G. H. Eijsink, J. Nissen-Meyer, Antagonistic activity of Lactobacillus plantarum C11: Two new two- peptide bacteriocins, plantaricins EF and JK, and the induction factor plantaricin A. *Appl. Environ. Microbiol.* **64**, 2269–2272 (1998).
105. D. D. Heeney, V. Yarov-Yarovoy, M. L. Marco, Sensitivity to the two peptide bacteriocin plantaricin EF is dependent on CorC, a membrane-bound, magnesium/cobalt efflux protein. *Microbiologyopen*. **8**, e827 (2019).
106. B. Ekblad, J. Nissen-Meyer, T. Kristensen, Whole-genome sequencing of mutants with increased resistance against the two-peptide bacteriocin plantaricin JK reveals a putative receptor and potential docking site. *PLoS One*. **12**, e0185279 (2017).
107. C. Oppegård, M. Kjos, J. W. Veening, J. Nissen-Meyer, T. Kristensen, A putative amino acid transporter determines sensitivity to the two-peptide bacteriocin plantaricin JK. *Microbiologyopen*. **5**, 700–708 (2016).
108. S. Iwatani, N. Ishibashi, F. P. Flores, T. Zendo, J. Nakayama, K. Sonomoto, LnqR, a TetR-family transcriptional regulator, positively regulates lacticin Q production in *Lactococcus lactis* QU 5. *FEMS Microbiol. Lett.* **363**, fnw200 (2016).
109. K. Fujita, S. Ichimasa, T. Zendo, S. Koga, F. Yoneyama, J. Nakayama, K. Sonomoto, Structural analysis and characterization of lacticin Q, a novel bacteriocin belonging to a new family of unmodified bacteriocins of gram-positive bacteria. *Appl. Environ. Microbiol.* **73**, 2871–2877 (2007).
110. F. Yoneyama, Y. Imura, S. Ichimasa, K. Fujita, T. Zendo, J. Nakayama, K. Matsuzaki, K. Sonomoto, Lacticin Q, a lactococcal bacteriocin, causes high-level membrane permeability in the absence of specific receptors. *Appl. Environ. Microbiol.* **75**, 538–541 (2009).
111. M. Kleerebezem, Quorum sensing control of lantibiotic production; nisin and subtilin autoregulate their own biosynthesis. *Peptides*. **25** (2004), pp. 1405–1414.
112. O. McAuliffe, R. P. Ross, C. Hill, Lantibiotics: structure, biosynthesis and mode of action. *FEMS Microbiol. Rev.* **25**, 285–308 (2001).
113. E. Breukink, C. Van Kraaij, R. A. Demel, R. J. Siezen, O. P. Kuipers, B. De Kruijff, The C-terminal region of nisin is responsible for the initial interaction of nisin with the target membrane. *Biochemistry*. **36**, 6968–6976 (1997).
114. E. Breukink, I. Wiedemann, C. Van Kraaij, O. P. Kuipers, H. G. Sahl, B. De Kruijff, Use of the cell wail precursor lipid II by a pore-forming peptide antibiotic. *Science (80-.).* **286**, 2361–2364 (1999).
115. B. B. Bonev, E. Breukink, E. Swiezewska, B. De Kruijff, A. Watts, Targeting extracellular pyrophosphates underpins the high selectivity of nisin. *FASEB J.* **18**, 1862–1869 (2004).
116. T. Stein, S. Borchert, P. Kiesau, S. Heinzmann, S. Klöss, C. Klein, M. Helfrich, K.-D. Entian, Dual control of subtilin biosynthesis and immunity in Bacillus subtilis. *Mol. Microbiol.* **44**, 403–416 (2002).
117. M. Kleerebezem, R. Bongers, G. Rutten, W. M. D. Vos, O. P. Kuipers, Autoregulation of subtilin biosynthesis in Bacillus subtilis: the role of the spa-box in subtilin-responsive promoters. *Peptides*. **25**, 1415–1424 (2004).
118. C. Klein, C. Kaletta, N. Schnell, K. Entian, U. Schneider, F. Gotz, H. Zahner, R. Kellner, G. Jung, "Analysis of Genes Involved in Biosynthesis of the Lantibiotic Subtilin" (1992).
119. J. Parisot, S. Carey, E. Breukink, W. C. Chan, A. Narbad, B. Bonev, Molecular mechanism of target recognition by subtilin, a class I lanthionine antibiotic. *Antimicrob. Agents Chemother.* **52**, 612–618 (2008).
120. D. Wang, Q. Wang, Y. Qiu, C. T. Nomura, J. Li, S. Chen, Untangling the transcription regulatory network of the bacitracin synthase operon in Bacillus licheniformis DW2. *Res. Microbiol.* **168**, 515–523 (2017).

121. Y. M. Harel, A. Bailone, E. Bibi, Resistance to bacitracin as modulated by an Escherichia coli homologue of the bacitracin ABC transporter BcrC subunit from Bacillus licheniformis. *J. Bacteriol.* **181**, 6176–6178 (1999).
122. T. J. Pollock, L. Thorne, M. Yamazaki, M. J. Mikolajczak, R. W. Armentrout, Mechanism of bacitracin resistance in gram-negative bacteria that synthesize exopolysaccharides. *J. Bacteriol.* **176**, 6229–6237 (1994).
123. K. J. Stone, J. L. Strominger, Mechanism of action of bacitracin: complexation with metal ion and C 55 -isoprenyl pyrophosphate. *Proc. Natl. Acad. Sci. U. S. A.* **68**, 3223–3227 (1971).
124. S. Y. Park, S. K. Choi, J. Kim, T. K. Oh, S. H. Park, Efficient production of polymyxin in the surrogate host Bacillus subtilis by introducing a foreign ectB gene and disrupting the abrB gene. *Appl. Environ. Microbiol.* **78**, 4194–4199 (2012).
125. M. E. Falagas, S. K. Kasiakou, Colistin: The revival of polymyxins for the management of multidrug-resistant gram-negative bacterial infections. *Clin. Infect. Dis.* **40** (2005), pp. 1333–1341.
126. B. A. Newton, THE PROPERTIES AND MODE OF ACTION OF THE POLYMYXINS. *Microbiol. Mol. Biol. Rev.* **20** (1956).
127. M. Schindler, M. J. Osborn, Interaction of Divalent Cations and Polymyxin B with Lipopolysaccharide. *Biochemistry*. **18**, 4425–4430 (1979).
128. H. Paulus, E. Gray, The Biosynthesis of Polymyxin B by Growing Cultures of Bacillus polymyxa. *J. Biol. Chem.* **239**, 865–871 (1964).
129. P. Stragier, R. Losick, Molecular genetics of sporulation in Bacillus subtilis. *Annu. Rev. Genet.* **30** (1996), pp. 297–341.
130. S. Kies, C. Vuong, M. Hille, A. Peschel, C. Meyer, F. Götz, M. Otto, Control of antimicrobial peptide synthesis by the agr quorum sensing system in Staphylococcus epidermidis: Activity of the lantibiotic epidermin is regulated at the level of precursor peptide processing. *Peptides*. **24**, 329–338 (2003).
131. R. Kellner, G. Jung, T. Horner, H. Zahner, N. Schnell, K. D. Entian, F. Gotz, Gallidermin: a new lanthionine-containing polypeptide antibiotic. *Eur. J. Biochem.* **177**, 53–59 (1988).
132. H. Brötz, M. Josten, I. Wiedemann, U. Schneider, F. Götz, G. Bierbaum, H.-G. Sahl, Role of lipid-bound peptidoglycan precursors in the formation of pores by nisin, epidermin and other lantibiotics. *Mol. Microbiol.* **30**, 317–327 (1998).
133. L. Foulston, M. Bibb, Feed-forward regulation of microbisporicin biosynthesis in Microbispora corallina. *J. Bacteriol.* **193**, 3064–3071 (2011).
134. L. T. Fernández-Martínez, J. P. Gomez-Escribano, M. J. Bibb, A relA-dependent regulatory cascade for auto-induction of microbisporicin production in Microbispora corallina. *Mol. Microbiol.* **97**, 502–514 (2015).
135. F. Castiglione, A. Lazzarini, L. Carrano, E. Corti, I. Ciciliato, L. Gastaldo, P. Candiani, D. Losi, F. Marinelli, E. Selva, F. Parenti, Determining the Structure and Mode of Action of Microbisporicin, a Potent Lantibiotic Active Against Multiresistant Pathogens. *Chem. Biol.* **15**, 22–31 (2008).
136. G. Liu, K. F. Chater, G. Chandra, G. Niu, H. Tan, Molecular Regulation of Antibiotic Biosynthesis in Streptomyces. *Microbiol. Mol. Biol. Rev.* **77**, 112–143 (2013).
137. S. Mak, J. R. Nodwell, Actinorhodin is a redox-active antibiotic with a complex mode of action against Gram-positive cells. *Mol. Microbiol.* **106**, 597–613 (2017).
138. T. Haneishi, N. Kitahara, Y. Takiguchi, M. Arai, S. Sugawara, New antibiotics, methylenomycins A and B: I. producing organism, fermentation and isolation, biological activities and physical and chemical properties. *J. Antibiot. (Tokyo).* **27**, 386–392 (1974).
139. G. A. Idowu, Investigations into the biosynthesis and mode of action of methylenomycin antibiotics from Streptomyces coelicolor. (2017) (available at http://webcat.warwick.ac.uk/record=b3111820~S15).
140. G. Xu, J. Wang, L. Wang, X. Tian, H. Yang, K. Fan, K. Yang, H. Tan, “Pseudo” γ-butyrolactone receptors respond to antibiotic signals to coordinate antibiotic biosynthesis. *J. Biol. Chem.* **285**, 27440–27448 (2010).
141. R. K. Bhatnagar, J. L. Doull, L. C. Vining, Role of the carbon source in regulating chloramphenicol production by Streptomyces venezuelae: studies in batch and continuous

cultures. *Can. J. Microbiol.* **34**, 1217–1223 (1988).
142. G. Niu, K. F. Chater, Y. Tian, J. Zhang, H. Tan, Specialised metabolites regulating antibiotic biosynthesis in Streptomyces spp. *FEMS Microbiol. Rev.* **40**, 554–573 (2016).
143. A. S. Mankin, R. A. Garrett, Chloramphenicol resistance mutations in the single 23S rRNA gene of the archaeon Halobacterium halobium. *J. Bacteriol.* **173**, 3559 (1991).
144. F. Schlünzen, R. Zarivach, J. Harms, A. Bashan, A. Tocilj, R. Albrecht, A. Yonath, F. Franceschi, Structural basis for the interaction of antibiotics with the peptidyl transferase centre in eubacteria. *Nature*. **413**, 814–821 (2001).
145. P. G. S. Mortimer, L. J. V. Piddok, The accumulation of five antibacterial agents in porin-deficient mutants of escherichia coli. *J. Antimicrob. Chemother.* **32**, 195–213 (1993).
146. J. L. Burns, P. M. Mendelman, J. Levy, T. L. Stull, A. L. Smith, A permeability barrier as a mechanism of chloramphenicol resistance in Haemophilus influenzae. *Antimicrob. Agents Chemother.* **27**, 46–54 (1985).
147. Y. Ohnishi, H. Yamazaki, J. Y. Kato, A. Tomono, S. Horinouchi, AdpA, a central transcriptional regulator in the A-factor regulatory cascade that leads to morphological development and secondary metabolism in Streptomyces griseus. *Biosci. Biotechnol. Biochem.* **69** (2005), pp. 431–439.
148. L. Luzzatto, D. Apirion, D. Schlessinger, Mechanism of action of streptomycin in E. coli: interruption of the ribosome cycle at the initiation of protein synthesis. *Proc. Natl. Acad. Sci. U. S. A.* **60**, 873–880 (1968).
149. R. Nakae, T. Nakae, Diffusion of aminoglycoside antibiotics across the outer membrane of Escherichia coli. *Antimicrob. Agents Chemother.* **22**, 554 (1982).
150. I. Iscla, R. Wray, S. Wei, B. Posner, P. Blount, Streptomycin potency is dependent on MscL channel expression. *Nat. Commun. 2014 51*. **5**, 1–7 (2014).
